# Sphenoid sinus is a rare site for tumor-induced osteomalacia: A case report and literature review

**DOI:** 10.3389/fendo.2023.1116793

**Published:** 2023-03-24

**Authors:** Fen Wang, Wentao He, Delin Ma, Weijie Xu, Junhui Xie, Gang Yuan

**Affiliations:** ^1^ Department of Endocrinology, Tongji Hospital, Tongji Medical College, Huazhong University of Science and Technology, Wuhan, China; ^2^ Branch of National Clinical Research Center for Metabolic Diseases, Wuhan, China

**Keywords:** tumor-induced osteomalacia, sphenoid sinus, phosphaturic mesenchymal tumor, sinonasal, neurilemmoma

## Abstract

**Background:**

In this paper, we present a rare case of tumor-induced osteomalacia (TIO) and a literature review of this rare disease.

**Methods:**

A case of TIO of the isolated sphenoid sinus was reported. Furthermore, the clinical features of TIO in the sphenoid sinus and other sinonasal sinuses were also reviewed and summarized.

**Results:**

A 35-year-old man with muscle weakness and lower back pain came to the Department of Neurology. No obvious neurological disease was found; however, magnetic resonance imaging of the extremities accidentally showed a tumor in the axilla. Bone scintigraphy showed suspicious bone metastasis. Hypophosphatemia was neglected. Interestingly, 2-deoxy-2-[fluorine-18]fluoro-d-glucose positron emission tomography/computed tomography (^18^F-FDG PET/CT) detected a tumor in the axilla and another in the sphenoid sinus, but only the tumor in the sphenoid sinus had somatostatin receptor (SSTR) expression in 68-gallium 1,4,7,10-tetraazacyclododecane-1,4,7,10-tetraacetic acid octreotate (Ga-68 DOTATATE) PET/CT. The sphenoid sinus tumor was proven to be a phosphaturic mesenchymal tumor (PMT), and the phosphate levels returned to normal after surgery. The literature review showed only 17 cases of TIOs that occurred in the sphenoid sinus, with an average age of 43.3 ± 13.7 years. Only three cases of TIOs in the sphenoid sinus did not invade the nasal cavity or other paranasal sinuses, which could be identified as isolated sphenoid sinus diseases. We compared the clinical features of sphenoid TIOs with those of non-sphenoid sinonasal TIOs, and it was found that the concentration of 1,25-dihydroxy vitamin D in the group with sphenoid TIOs was much higher than that in the group with non-sphenoid sinonasal TIOs. A total of 153 cases of TIOs in the sinonasal sinus were reviewed. The ethmoid sinus was found to be the major site (64.7%), followed by the nasal cavity (50.3%), maxillary sinus (19.0%), frontal sinus (16.4%), and sphenoid sinus (11.8%). There were 66 patients (43.1%) who showed tumors invading more than one sinus. Most of the tumors (69.3%) were diagnosed as PMTs by pathology, followed by hemangiopericytoma (14.3%). Immunostaining was beneficial in the differential diagnosis of these tumors; however, larger sample sizes are needed for better accuracy.

**Conclusion:**

TIO in the sinonasal sinus, especially in the sphenoid sinus, is rare. Moreover, isolated sphenoid sinus disease can be easily misdiagnosed. When the clinical manifestation of osteomalacia is atypical, associating it with sphenoid sinus disease is even more difficult. Thus, TIO in the sphenoid sinus needs further exploration.

## 1 Introduction

Hypophosphatemia osteomalacia is characterized by hypophosphatemia, high bone alkaline phosphatase (ALP) levels, muscle weakness, and bone pain. It is usually caused by severe vitamin D deficiency, hereditary hypophosphatemic rickets syndrome, tumor-induced osteomalacia (TIO), primary renal tubular defects, and drugs (1). TIO is a rare disease characterized by high levels of fibroblast growth factor 23 (FGF23). FGF23 is an important phosphatonin that promotes the loss of urinary phosphate *via* sodium phosphate cotransporters 2a and 2c and inhibits the 1α hydroxylase enzyme (2). Apart from inducing hypophosphatemia osteomalacia, FGF23 can also decrease the concentration of 1,25-dihydroxy vitamin D (3).

TIO is usually caused by a type of mesenchymal tumor at the morphological level. Weidner et al. named this tumor phosphaturic mesenchymal tumor (PMT) (4). PMTs are usually located in the lower extremities, followed by the head, pelvis or hip area, the thorax, and the upper extremities. Most PMTs that occur in the head are initiated in the nasal cavity, mandible, and lower gingiva. A few PMTs occur in the sphenoid sinus. Moreover, PMTs rarely exist with other types of tumors, such as neurilemmomas. In the present study, we report a rare case of a PMT located in the sphenoid sinus that coexisted with an axillary neurilemmoma. We also review the relevant literature.

## 2 Materials and methods

### 2.1 Case description

**Figure 1:**
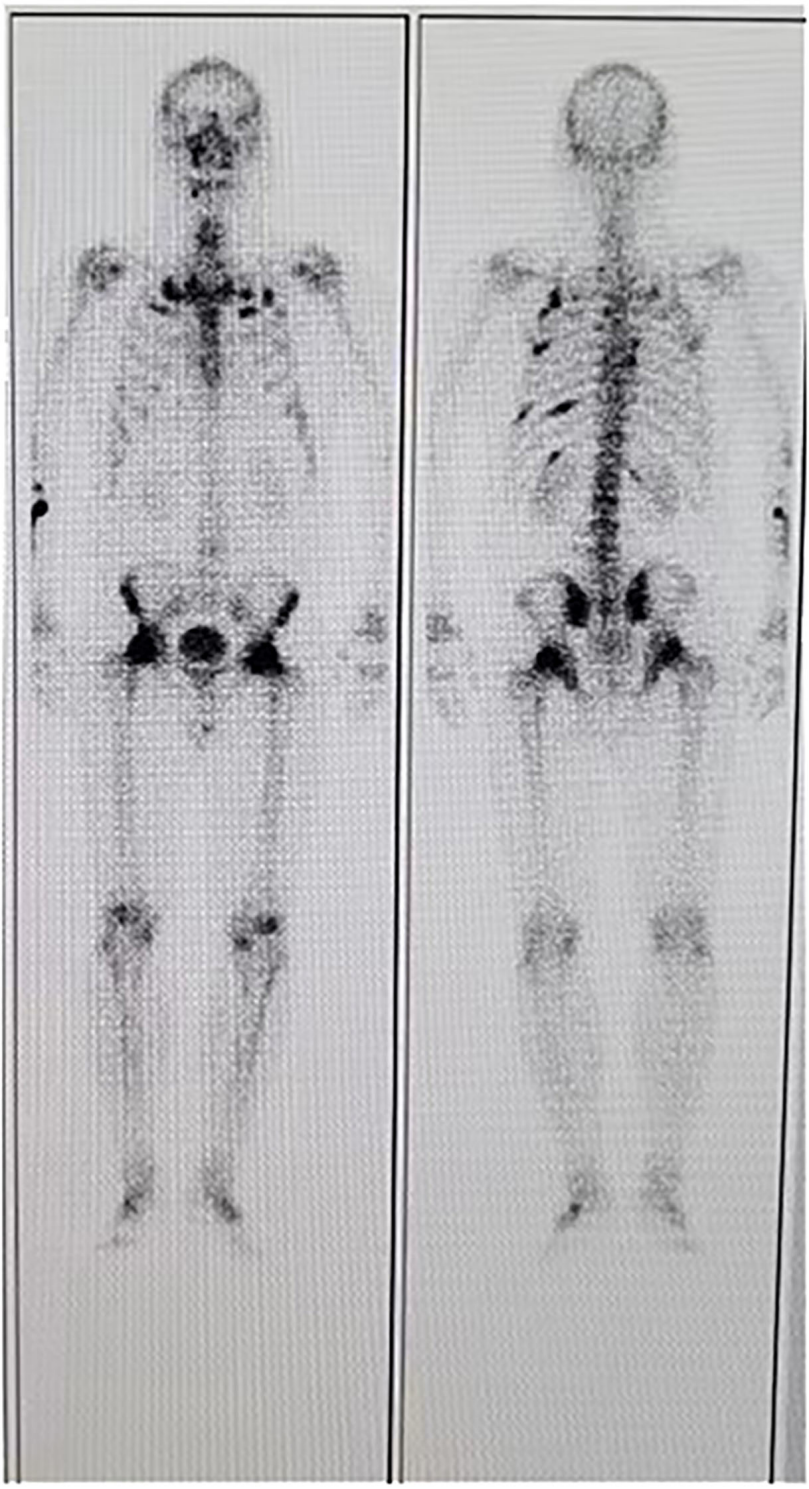
Bone scan of the patient showing multiple uptakes similar to metastasis.

**Figure 2:**
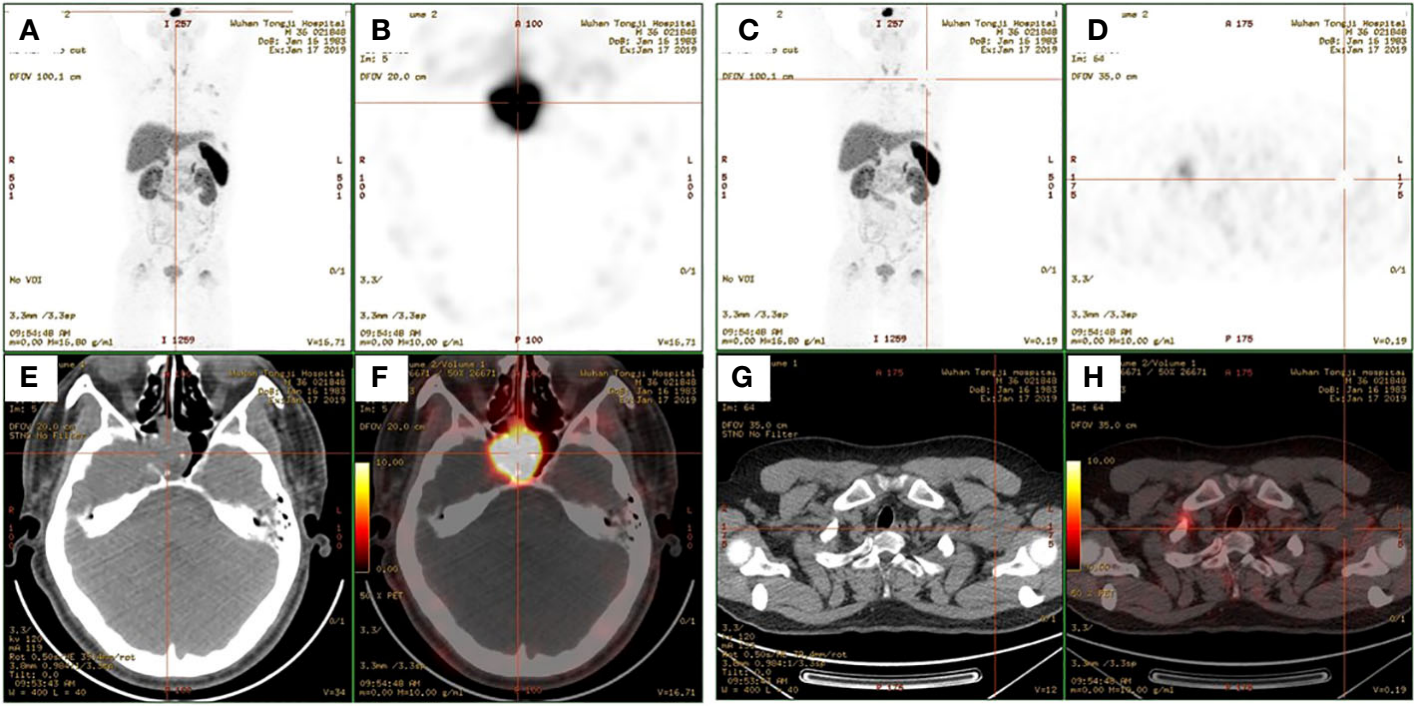
**(A, C)** Maximum-intensity projection images of the patient. **(B, D)** Axial views of the sphenoid sinus tumor **(B)** and the axillary tumor **(D)** in 68-gallium 1,4,7,10-tetraazacyclododecane-1,4,7,10-tetraacetic acid octreotate (Ga-68-DOTATATE) positron emission tomography (PET). **(E, G)** Axial views of the sphenoid sinus tumor **(E)** and the axillary tumor **(G)** in CT. **(F, H)** Fusion images.

**Figure 3:**
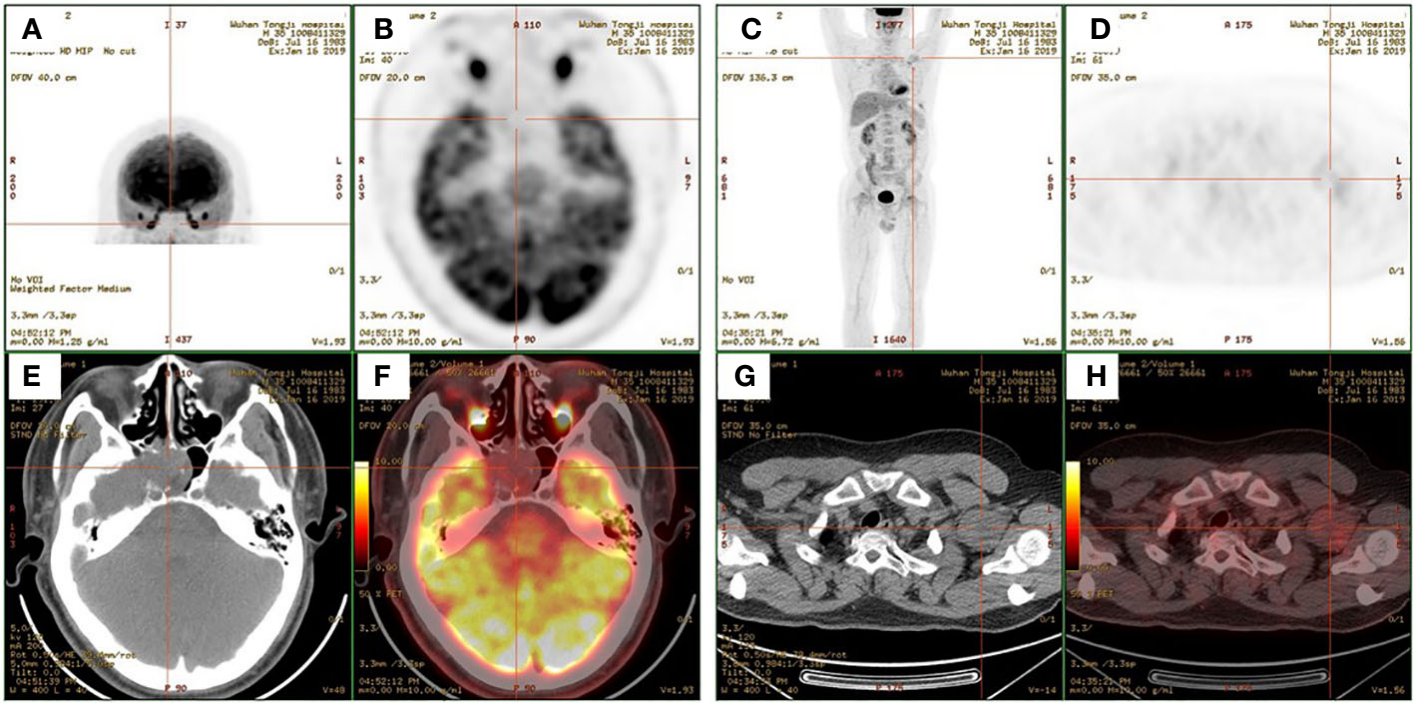
**(A, C)** Maximum-intensity projection images of the patient. **(B, D)** Axial views of the sphenoid sinus tumor **(B)** and the axillary tumor **(D)** in 2-deoxy-2-[fluorine-18]fluoro-d-glucose positron emission tomography (^18^F-FDG PET). **(E, G)** Axial views of the sphenoid sinus tumor **(E)** and the axillary tumor **(G)** in CT. **(F, H)** Fusion images.

A 35-year-old man came to the Department of Neurology with the main complaint of gradually aggravating muscle weakness and lower back pain for 1 year. He presented no headache, vomiting, and dystaxia and had a family history of similar diseases. Physical examination showed symmetrically impaired limb muscle strength with normal muscle tone. No dysphonia, dysphagia, paraesthesia, diplopia, and altered consciousness were found. His electromyogram report was normal. Magnetic resonance imaging (MRI) of the lower and upper extremities revealed fat deposition in the lower extremity muscles and a tumor in the axilla. No nerve compression or obvious inflammation was found in the lumbar spine. Therapy to relieve the pain was suggested for a temporary period. Due to continuous hypophosphatemia ranging from 0.28 to 0.49 mmol/L (normal range, 0.81–1.45 mmol/L), the patient was transferred to the Department of Endocrinology. His urine phosphate level was as high as 27 mmol/day, whereas his serum phosphate level was only 0.49 mmol/L. Furthermore, the patient’s ALP level was 171 U/L (normal range, 40–130 U/L). The serum calcium and urine calcium concentrations were normal. His parathyroid hormone (PTH) level was 66.72 pg/ml (normal range, 15–65 pg/ml), and his vitamin D3 level was 22.4 ng/ml. Dual-energy X-ray absorptiometry revealed *z*-scores of −3.0, −2.0, and −1.8 for the left femoral neck, left hip, and lumbar region, respectively. Bone scintigraphy showed suspicious bone metastasis due to multiple bilateral uptakes in the ribs, hip joint, knee, proximal femur, and ankle (**Figure 1**). However, hypophosphatemia osteomalacia was suspected as the first diagnosis. The 18-Fluoride-fluorodeoxyglucose positron emission tomography/computed tomography (^18^F-FDG PET/CT) showed a tumor in the axilla (47 × 37 mm) and a tumor in the sphenoid sinus (30 × 26 mm) with a maximum standard uptake value (SUV_max_) of 3.0 (**Figures 2**, **3**). Only the tumor in the sphenoid sinus showed prominent somatostatin receptor (SSTR) gene expression as shown by the 68-gallium 1,4,7,10-tetraazacyclododecane-1,4,7,10-tetraacetic acid octreotate (Ga-68-DOTATATE) PET/CT (SUV_max_ = 30.2). TIO was diagnosed with a culprit tumor in the sphenoid sinus. The patient underwent a resection of the sphenoid sinus tumor. His serum phosphate concentration increased to 1.13 mmol/L in the first follow-up after the surgery. Pathology diagnosis showed a PMT with the negative immunohistochemistry of S-100. A month later, the patient underwent a resection of the axillary tumor. Histological analyses revealed a neurilemmoma with the positive immunohistochemistry of S-100.

### 2.2 Literature review

Sinonasal TIOs include TIOs that occur in the nasal cavity, ethmoid sinus, frontal sinus, maxillary sinus, and sphenoid sinus. Relevant studies were collected using the PubMed database to review the sphenoid and sinonasal TIOs reported in the literature. The following keywords were used: “mesenchymal tumor,” “hypophosphataemic osteomalacia,” “phosphaturic mesenchymal tumor,” “mesenchymal phosphaturic tumor,” “oncogenic osteomalacia,” “tumor-associated hypophosphataemic osteomalacia,” and “tumor-induced osteomalacia” in combination with “nasal, sinonasal, or paranasal sinuses” or “nasal, sphenoid, sinus or ethmoid sinus,” or “nasopharyngeal, craniofacial, or head.” In addition, the end-list references in the case studies and reviews were also checked to include all relevant cases. We tried to find all the original papers to check the information in similar reviews, although several case reports failed to locate the original papers. The last literature search was performed on April 5, 2022.

### 2.3 Data collection

We collected information on the age, gender, clinical features, duration of disease, tumor site, multiple sinonasal invasion, the levels of serum phosphate, serum ALP, PTH, 1,25-dihydroxy vitamin D, and FGF23, the biopsy of the tumor, functional scintigraphy, immunostaining, recurrence, and the outcomes. The clinical features included muscle symptoms such as weakness; osteomalacia-associated symptoms such as bone pain, fracture, difficulty in walking, and shortening of height, among others; and symptoms caused by the tumor location such as nasal obstruction, headache, and epistaxis. The course of the disease was calculated from the date of symptom onset to the first operation. A local invasion was defined as tumors extending to the adjacent sinonasal sinus, intracranial fossa, and orbit; adjacent soft tissues such as the nasopharynx; and adjacent fossa such as the pterygopalatine fossa and infratemporal fossa. If multiple preoperative serum phosphates were recorded, the average value was adopted. Since the normal ranges for ALP, PTH, and FGF23 varied between the different reports, we converted these values as times for the upper limit of normal (ULN). If no reference range was given in the paper, the value in each column was substituted as normal, high, or low.

### 2.4 Statistical analysis

Data were analyzed using GraphPad software. The Shapiro–Wilk test was used to determine whether the data were normally distributed. Continuous variables were expressed as the mean ± SD for data with normal distribution, while the median and interquartile range (IQR) were used for data with non-normal distribution. Normally distributed data were subjected to Student’s *t*-test; otherwise, the Mann–Whitney *U* test was used. Fisher’s exact test was performed for categorical variables. A *p*-value <0.05 was considered statistically significant.

## 3 Results

### 3.1 TIOs in the sphenoid sinus

Only 17 cases (including our study) of TIOs in the sphenoid sinus were obtained. The detailed clinical features of these patients are presented in **Table 1** (5–16). The ratio of male cases to female cases for sphenoid sinus TIOs was 1.83:1. The average age of these patients was 43.3 ± 13.7 years. The majority (16/17) of these cases had muscle symptoms or osteomalacia, while at least six cases showed local symptoms such as nasal obstruction, headache, and epistaxis. Case 4 showed no muscle or osteomalacia-associated symptoms and had normal ALP and PTH levels; however, the preoperative serum phosphate level was absent. Only three cases (including our case) showed sphenoid sinuses without invasion in the nasal cavity, other paranasal sinuses, intracranial fossa, orbit, and adjacent fossa, which could be identified as isolated sphenoid sinus diseases. Case 16 reported a recurrence that occurred 32 months after the first surgery. TIO was cured in the majority (16/17) of these cases, while the remaining case did not report the outcome.

**Table 1:** Clinical features, diagnosis, and outcomes of tumor-induced osteomalacias (TIOs) in the sphenoid sinus.

	Author, year	Sex/age (years)	Hypophosphatemia-associated symptom	Local symptom	Serum phosphate (mmol/l)	ALP (U/L)	1,25-Dihydroxy vitamin D (pg/ml)	Serum PTH^a^	Serum FGF23^a^	Immunohistochemistry	FGF23 expression in tumor	Duration of disease (month)	Tumor site	Histological diagnosis	Tumor invasion	Treatment	Outcome and recurrence
1	Battoo et al., 2010 (5)	F/34	Yes	Nasal blockage and epistaxis	0.42	1823	55.8	1.26	NA	Positive for NSE (+). Negative for CgA, leukocyte common antigen, cytokeratin, CD34, Syn, CD99		72	Left maxillary sinus, nasal cavity, ethmoid sinuses, nasal septum, medial wall of both maxillary antra, the medial wall of both orbits, and nasal roof	Giant cell tumor	Local invasion	Surgery and adjuvant radiotherapy	Cured, NED (36 months)
2	Catalano et al., 1996 (6)	F/66	Yes	Nasal obstruction, frontal headache, and epistaxis	0.58	222	NA	Normal	NA	Positive for vimentin, muscle cell antigen and FVIII		many years	Right maxillary, ethmoid sinus, cribriform plate, and sphenoid sinus	Hemagiopericytoma	Local invasion	Tumor embolization and surgery	Cured, NED (25 months)
3	Chang et al., 2012 (7)	M/42	Yes	Epistaxis and nasal obstruction	0.51	–	26	0.82	–	–	Positive	60	Left nasal cavity, ethmoid sinus, invading walls from the left maxillary sinus, sphenoid bone, and lamina papyracea	Hemangiopericytoma	Local invasion	Surgery	NA
4	Deep et al., 2014 (8)	M/41	No	Nasal obstruction, discolored rhinorrhea, anosmia, dysgeusia, and facial pressure	–	Normal	Normal	Normal	–	–	–	NA	Nasal septum, basisphenoid, and ethmoid roof	PMT (no osteomalacia)	Local invasion	Surgery	Cured, NED (24 months)
5	Folpe et al., 2004 (9)	M/21	Yes	NA	–	–	–	–	–	–	–	24	Ethmoid and sphenoid sinus	Hemangiopericytoma-like tumor	Local invasion	Surgery	Cured, NED (24 months)
6	Guglielmi et al., 2011 (10)	M/22	Yes	NA	–	–	–	–	–	–	Positive	24	Rhinopharynx, left ethmoid, and sphenoid sinus	Hemangiopericytoma-like tumor	Local invasion	Resurgery^b^	Cured, NED (NA)
7	Gupta et al., 2009 (11)	M/51	Yes	NA	0.39	2943	–	Normal		Positive for vimentin. Negative for S-100, desmin, SMA, and cytokeratin	–	108	Nasal cavity, ethmoid sinus, and sphenoid sinus	PMT	Local invasion	Surgery	Cured, NED (NA)
8	Kurien et al., 2019 (12)	M/55	Yes	NA	0.55	383	–	1.11	–	Positive for vimentin, SMA, CD99, desmin, and CD34. Negative for S-100 and CD68	–	24	Ethmoid and sphenoid sinus	Hemangiopericytoma, revised as PMT	Local invasion	Surgery	Cured, NED (36 months)
9	Kurien et al., 2019 (12)	F/62	No	Epistaxis and nasal blockage	–	–	–	–	–	Positive for SMA. Negative for CD34, STAT6, EMA, and β-catenin	–	48	Nasal cavity, maxillary sinus, anterior ethmoid, posterior ethmoid, and sphenoid	PMT	Local invasion	Surgery	Cured, NED (3 months)
10	Linsey et al., 1983 (13)	F/54	Yes	Epistaxis	0.52	151	–	–	–	–	–	30	Ethmoid, maxillary, and sphenoid sinus	Angiofibroma, revised as PMT	Local invasion	Surgery	Cured, NED (NA)
11	Ungari et al., 2004 (14)	M/24	Yes	No	0.48	603	62	1.34	1.19	–	–	NA	Ethmoid and sphenoid sinus	PMT	Local invasion	Resurgery^b^	Cured, NED (24 months)
12	Raj et al., 2021 (15)	F/39	Yes	NA	0.48	603	62	1.34	1.19	Positive for S100. Negative for CD34, CK, SMA, LCA, SAL4, and caldesmin	–	8	Right frontal, ethmoid, maxillary, and sphenoid sinuses and expanding into the right nasal cavity	Glomus tumor	Local invasion	Surgery	Cured, NED (3 months)
13	Zhu et al., 2021 (16)	M/54	Likely Yes	Not specific	–	–	–	–	–	–	–	84	Sphenoid sinus	PMT	No	Surgery	Cured, NED (57 months)
14	Zhu et al., 2021 (16)	M/50	Likely Yes	Not specific	–	–	–	–	–	–	–	12	Sphenoid sinus and ethmoid sinus	PMT	Local invasion	Surgery	Cured, NED (12 months)
15	Zhu et al., 2021 (16)	F/52	Likely Yes	Not specific	–	–	–	–	–	–	–	36	Sphenoid sinus	PMT	No	Surgery	Cured, NED (111 months)
16	Zhu et al., 2021 (16)	M/34	Likely Yes	Not specific	–	–	–	–	–	–	–	12	Sphenoid sinus and ethmoid sinus	PMT	Local invasion	Resurgery	Recurred 32 months later after the first surgery, NED (26 months) after second surgery
17	This case	M/35	Yes	No	0.49	171	–	1.03	–	Positive for SATB2, FLI1, CD56, and cathepsin K. Negative for S-100, EMA, CD34, GFAP, PR, CD31, ERG, desmin, HMB45, Melan-A, SOX10, PCK, Syn, DOG1, and β-catenin	–	12	Sphenoid sinus with local invasion of sellar floor	PMT	No	Surgery	Cured, NED (24 months)

En dash indicates that the value is not available.

ALP, alkaline phosphatase; CD, cluster of differentiation; CK, cytokeratin; EMA, epithelial membrane antigen; ERG, E26 transformation-specific transcription factor; FGF23, fibroblast growth factor 23; Fli-1, Friend leukemia integration-1; GFAP, glial fibrillary acidic protein; LCA, leukocyte common antigen; HMB45, human melanoma black 45; MelanA, melanoma antigen recognized by T cell; NA, not available; NED, no evidence of disease; Not specific, the case series did not report the individual data in this patient; PCK, pan-cytokeratin; PR, progesterone receptor; SATB, special AT-rich sequence binding protein; SMA, smooth muscle actin; SOX10, SRY-box transcription factor 10; STAT6, signal transducer and activator of transcription 6; Syn, synaptophysin; PMT, phosphaturic mesenchymal tumor.

a The values in this column were defined as multiples of the upper limit of normal. ^b^The first surgery was not complete.

### 3.2 TIOs in the sinonasal sinus

To summarize, 153 cases of sinonasal TIOs were reported, including our case (**Supplementary Material**). Among them, 74 were male cases and 78 were female cases. The gender of one case was not reported. The ratio of male cases to female cases was 1:1.05, and the average age at diagnosis was 45.2 ± 11.2 years. Most of the sinonasal TIOs (124/153, 81.0%) were diagnosed in patients aged from 30 to 60 years. A total of 16 patients (10.5%) were aged 60 years or older, nine patients (5.9%) were in their 20s, and two patients (1.3%) were diagnosed in their teenage years. Age was not reported in two cases (1.3%). TIOs in the sinonasal sinus have often been reported as occupying adjacent sinuses, bones, or soft tissues. Ethmoid sinus was the predominant site (64.7%), followed by the nasal cavity (50.3%), maxillary sinus (19.0%), frontal sinus (16.4%), sphenoid sinus (11.8%), intracranial fossa (5.9%), orbit (2.6%), nasopharynx (2.6%), and adjacent fossa (1.3%). A total of 66 patients (43.1%) showed tumors invading more than one sinus. Among the 86 TIOs that were present in only one sinus, 45.3% occurred in the ethmoid sinus, followed by the nasal cavity (30.2%), maxillary sinus (14.0%), frontal sinus (7.0%), and sphenoid sinus (3.5%).

### 3.3 Comparison of the clinical features between sphenoid TIOs and non-sphenoid sinonasal TIOs

We compared the clinical features of sphenoid TIOs with those of non-sphenoid sinonasal TIOs (**Table 2**). The differences in the age, gender distribution, duration of disease, serum phosphate, and the ALP, PTH, and FGF23 levels between the two groups were not significant. However, the concentration of 1,25-dihydroxy vitamin D in the sphenoid TIO group was much higher than that in the non-sphenoid sinonasal TIO group. Most papers reported the clinical symptoms associated with hypophosphatemia, except for one paper. In addition, two case series did not show the clinical symptoms linked with hypophosphatemia for each patient. Although these patients in the two case series were likely to have clinical symptoms linked with hypophosphatemia, we could not confirm. It is interesting to note that five cases reported no symptoms associated with hypophosphatemia. A total of 16 patients were cured in the sphenoid group, but the outcome of one patient was unknown. In the non-sphenoid sinonasal group, 118 cased were cured.

**Table 2:** Clinical features of the different groups.

	All TIOs	Sphenoid sinus TIOs	Non-sphenoid sinonasal TIOs	*p*-value
*N*	153	17	136	
Age (years)	45.2 ± 11.2	43.3 ± 13.7	45.5 ± 10.9	>0.05
Men-to-women ratio	74:78	11:6	63:72^b^	>0.05
Duration of disease (months)	36.0 (24.0–60.0)	27.0 (12.0–63.0)	36.0 (24.0–60.0)	>0.05
Clinical symptoms				
Without osteomalacia	5	1	4	>0.05
Local symptom	46	6	40	>0.05
Biochemical results				
Serum phosphate (mmol/l)	0.49 ± 0.13, *n* = 79	0.48 ± 0.07, *n* = 9	0.49 ± 0.14, *n* = 70	>0.05
ALP (IU/L)	300.0 (175.5–431.0), *n* = 61	351.0 (183.8–1518.0), *n* = 8	293.0 (175.5–425.5), *n* = 53	>0.05
1,25-Dihydroxy vitamin D (pg/ml)	13.0 (7.3–22.0), *n* = 27	55.8 (26.0–62.0), *n* = 3	11.7 (7.1–20.4), *n* = 24	0.0252
PTH^a^	0.95 (0.71–1.36), *n* = 39	1.11 (0.93–1.30), *n* = 5	0.93 (0.69–1.39), *n* = 34	>0.05
FGF23^a^	3.68 (2.21–7.77), *n* = 33	1.19, *n* = 1	4.00 (2.42–7.83), *n* = 32	
PMT diagnosis, *n* (%)	106 (69.3%)	11 (64.7%)	96 (69.9%)	>0.05
Outcome				
Recurrence	11	1	10^c^	
Cured	134	16	118	>0.05
Died	5	0	5	
NA or not cured	14	1	13	

TIO, tumor-induced osteomalacia; ALP, alkaline phosphatase; PTH, parathyroid hormone; FGF23, fibroblast growth factor 23; PMT, phosphaturic mesenchymal tumor; NA, not available.

a The values in these rows were calculated as times the upper limit of normal.

b The gender of one case was unknown.

c Three patients had recurrence and died and seven patients were alive.

### 3.4 Biopsy and functional scintigraphy of TIOs in the sinonasal sinus

The results of the biopsy of the tumor were reported in 22 cases. The pathological results of the biopsy in 11 patients were consistent with the final pathological results of the resected tumor. However, 50% of the biopsy specimens were insufficient for diagnosis or led to a different diagnosis. After the prevalence of functional scintigraphy, the diagnosis of TIOs became much easier. There were 53 cases that reported positive scanning of octreotide scintigraphy, while 39 cases reported positive results in ^18^F-FDG PET/CT or the somatostatin receptor PET/CT.

### 3.5 Therapy, recurrence, and outcome of TIOs

Most of the cases reported in the literature were cured by surgery, embolism, medication, radiotherapy, or a combination of these therapies (**Table 3**). A total of 21 patients received more than one surgery due to recurrence or incomplete surgery. One patient underwent two surgeries for two culprit tumors. Most patients showed good outcomes, with 111 cases reported to have normalized phosphate levels 1 day to 1 month after surgery. Five patients died of cerebral hernia, bronchopneumonia, cerebral hemorrhage before surgery, epistaxis, and metastasis of colon cancer. Nine cases reported intracranial invasion, two cases died, and five cases were alive with no evidence of disease. One case was diagnosed as malignant PMT without a long-term outcome, while the outcome of one case was unavailable.

**Table 3:** Summary of the treatment and outcomes of the 153 cases.

Treatment	*N*	Outcome
Surgery	129	NED (122), recurrence with unknown outcome (1), died of brain hernia (1), NA (5)
Surgery + radiotherapy	11	NED (7), alive with disease (1), NA (1), tumor remains with normal phosphate at 30 months (1), died of epistaxis (1)
Surgery + medication	4	NED (1), not cured (2), recurrence and died of colon cancer (1)
Surgery + embolization	3	NED (2), NA (1)
Surgery + embolization + radiotherapy	1	NED (1)
Surgery + embolization + radiotherapy + medication	1	Recurrence at 3 months and died of bronchopneumonia (1)
Medication	2	NA (1), died of cerebral hemorrhage before surgery (1)
NA	2	NA (1), recurrence (1)

NA, not available; NED, no evidence of disease.

### 3.6 Pathology and immunostaining of TIOs

Although PMTs are the mainstream pathological diagnosis in patients with TIO, almost 30.7% of tumors are still diagnosed as other tumors, including hemangiopericytoma, glomangiopericytoma, and glomangioma (**Table 4**), among others. Four cases with other pathology diagnoses were revised as PMT according to an updated diagnosis or a suggested diagnosis by Folpe et al. (9, 12, 17). Histopathological results are not specific in PMTs; however, some of the immunostaining results may help in the differential diagnosis. We summarized the immunohistochemical results in the review, including our case, in **Table 5**. All the reported cases had positive immunostaining of SSTR, CD56, vimentin, and special AT-rich sequence-binding protein 2 (SATB2). Furthermore, all the reported cases had negative immunostaining of synaptophysin (Syn), chromogranin, AE1/AE3, and epithelial membrane antigen (EMA). The majority of the immunostaining results for desmin, S-100, and CD34 were negative. The immunostaining results for smooth muscle actin (SMA), CD99, neuron-specific enolase (NSE), and CD68 were not consistent. The expression of FGF23 in tumors is a strong indicator of TIOs. Only 12 cases reported the expression of FGF23 through immunostaining, mRNA expression, chromogenic *in-situ* hybridization, or Northern hybridization of FGF23 in tumors or tumor-derived cell lines.

**Table 4:** Histopathological diagnosis of the 153 cases assessed in the present study.

Histopathological diagnosis	*N*
PMT	106 (including 1 malignant PMT)
Hemangiopericytoma	22
Hemangiopericytoma-like tumor	5
Glomangiopericytoma	4
Glomangioma	2
Glomus tumor	2
Ossifying fibromyxoid tumor	2
Odontogenic fibroma	1
Odontogenic tumor	1
Angiofibroma	1
Arteriovenous hemangioma	1
Giant cell tumor	1
Hemangiofibroma	1
Malignant schwannoma	1
Mesenquimal tumor	1
NA	2

NA, not available; PMT, phosphaturic mesenchymal tumor.

**Table 5:** Summary of the immunohistochemical results in the review including our case.

	S-100	Syn	Chromogranin	AE1/AE3	SSTR	CD56	Vimentin	SATB2
Total	32	6	5	14	4	5	14	5
Positive	4	0	0	0	4	5	14	5
Negative	28	6	5	14	0	0	0	0
	SMA	CD99	NSE	Bcl-2	CD68	EMA	CD34	Desmin
Total	26	4	6	5	4	10	26	20
Positive	13	1	4	4	2	0	5	1
Negative	13	3	2	1	2	10	21	19

Syn, synaptophysin; SSTR, somatostatin receptor; SATB2, special AT-rich sequence-binding protein 2; SMA, smooth muscle actin; NSE, neuron-specific enolase; EMA, epithelial membrane antigen.

### 3.7 Multifocal TIOs and tumor coexisting with TIO

TIO is rarely caused by more than one tumor. However, Peterson et al. reported a case with a second PMT in the maxillary sinus 2 years after the first surgery of PMT in the tibia (**Supplementary Material**) (18). Both Arai et al. (19) and Higley et al. (20) also reported more than one culprit tumor in two different sites. Furthermore, PMT also rarely coexists with another type of tumor. However, Ha et al. reported a PMT case with concurrent lymphoma, wherein Ga-68-DOTATATE PET/CT played a crucial role in distinguishing the culprit tumor (21).

## 4 Discussion

In this study, we reported a rare case of oncogenic osteomalacia induced by a tumor at the sphenoid sinus and summarized the clinical features of TIOs occurring in the sphenoid sinus. Hypophosphatemia is common in patients. However, hypophosphatemia osteomalacia is a rare disease. In 2015, many researchers from Japan put forward a consensus on the diagnosis of hypophosphatemia osteomalacia (22). The manifestation was nonspecific, especially when the skeletal deformities and bone pain were mild. Misdiagnosis of hypophosphatemia osteomalacia is common. One study showed a mean delay of about 5 years before diagnosis (4). Occasionally, locating the culprit tumor in TIO is difficult. Owing to the application of SSTR scintigraphy or PET/CT, the detection rate of TIO has improved greatly. DOTATATE has a great affinity to SSTR2, which is abundantly expressed in PMT. Zhang et al. (23) reported that DOTATATE PET/CT showed a sensitivity of 100% and a specificity of 90.6% in detecting TIOs. A sensitivity of 100% is higher than that in clinical experience, but PET/CT of the somatostatin receptor, such as Ga-68-DOTATATE PET/CT, has been the most successful method used to locate the culprit tumor. Recently, a meta-analysis has shown a pooled sensitivity of 90% [95% confidence interval (CI) = 82%–95%] in Ga-DOTA–somatostatin PET/CT and 83% (95% CI = 75%–89%) in octreoscan–single-photon emission CT (SPECT)/CT (24).

The average age at diagnosis of sinonasal TIO was in the 30s to 50s, which was consistent with previous reviews of undefined TIOs (25, 26). The gender distribution was also similar to the general gender distribution of TIOs (25, 26). TIO can be caused by tumors located anywhere in the body. A recent review study indicated that TIOs in the head and neck accounted for 25.7% of the overall TIOs (27). Sinonasal TIOs constituted 33.3% of the overall TIOs in the head (26) or 61.9% of the overall TIOs in the head and neck (28). Different from other patients with TIO, who have no symptoms associated with tumor expansion or growth, those with sinonasal TIO may experience epistaxis and nasal obstruction (29), which can be found by physical examination or nasopharyngoscopy, which is easy to perform. However, the clinical manifestations of the isolated sphenoid are occult. Isolated sphenoid sinus tumors are usually diagnosed by CT or MRI rather than physical examination or nasopharyngoscopy. Isolated sphenoid sinus disease is an overlooked disease. A previous review study including 1,442 patients with isolated sphenoid disease indicated that the most common symptoms were headache (63.9%), followed by visual loss (35.3%), facial pain (31.2%), rhinorrhea (24.6%), diplopia (18.9%), fever (16.4%), and meningeal sign (4.9%) (30). Inflammatory diseases accounted for 75.0% of the isolated sphenoid diseases, followed by tumor disease (fibro-osseous lesions, 18.9%) and miscellaneous disease (6.1%) (30). TIO caused by isolated sphenoid tumors is rare. Among the 17 sphenoid TIO cases, 14 cases invaded the adjacent paranasal sinus. Only three cases were caused by isolated sphenoid tumors. Furthermore, in a study by Zhu et al., detailed information on two cases was not included (16).

Weidner et al. coined the pathological diagnosis of PMT in 1991 (4). In 1987, Weidner et al. (31) reported that PMTs can be classified into the following four categories: mixed connective tissue variant (PMTMCT), osteoblastoma-like variant, non-ossifying fibroma-like variant, and ossifying fibroma-like variant. PMTMCT, the most common variant, is composed of a distinctive admixture of small and round to spindled mesenchymal cells, along with prominent blood vessels, patchy calcifications, and hemangiopericytic cells, and shows some similarities to other tumors. Wu et al. proposed a fifth subtype, which is PMT of mixed epithelial and connective tissue type (PMTMECT) (32). However, the distinct pathological diagnosis of PMT is insufficient even after 31 years since its first definition. Flope et al. (9) reviewed 29 cases of oncogenic osteomalacia and three cases with similar histological features to PMT. Subsequently, 11 tumors were revised as PMTs, whose primary diagnoses were mesenchymal tumor (*N* = 2), giant cell tumor (*N* = 2), hemangiopericytoma (*N* = 1), osteosarcoma (*N* = 2), mesenchymal chondrosarcoma (*N* = 1), sarcoma (*N* = 1), spindle cell lipoma (*N* = 1), and sclerosing hemangioma (*N* = 1). Another three cases including two mesenchymal tumors and one chondroblastoma were revised as probable PMTs (9). Wu et al. (32) also analyzed 22 TIOs and reported that five cases with different original diagnoses (three odontogenic fibromas, one giant cell reparative granuloma, and one myofibroblastic tumor) should be revised as PMTs. There are another three cases (one giant cell tumor, one giant cell granuloma, and one ameloblastic fibrosarcoma) in the literature that would fit the diagnosis of PMT (32). A part of mesenchymal tumors associated with osteomalacia was diagnosed as other similar tumors. Differential diagnosis between PMT and its histological mimics is difficult. Immunohistochemistry features may help in the differential diagnosis. PMTs usually have positive immunostaining of FGF23 and somatostatin receptor 2A (SSTR2A). Negative staining of FGF23 and SSTR2A is important for ruling out the diagnosis of PMT. However, the applications of these two markers are restricted in many countries. Staining of other markers such as SMA, CD34, CD68, NSE, AE1/AE3, and Bcl-2 is not consistent in studies. Wu et al. (32) reported that the typical polyimmunophenotypic pattern (positive staining for AE1/AE3, vimentin, SSTR2A, FGF23, NSE, CD99, CD56, Bcl-2, and D2-40) is crucial for the differential diagnosis between the proposed PMTMECT type and its histological mimics (33). However, the application of CD99, Bcl-2, NSE, and D2-40 in PMT requires more evidence (32). The 100% positive staining of AE1/AE3 and NSE in their results was not consistent with our results. Their proposed PMTMECT subtype accounted for the opposite staining of AE1/AE3 because its positive staining was restricted in the epithelial cell components other than the mesenchymal cell components (32, 33). The reason for the inconsistent staining of NSE requires more study. Most cases showed negative immunostaining of S-100 and Syn, which was consistent with our results. S-100 is a marker of neuroendocrine tumors. Chatterjee et al. reported that an immunophenotype of PMT (SATB2^+^/ERG^+^/CD56^+^/S-100^−^/STAT6^−^) helped distinguish PMT from its histological mimics (34). We believe that a negative staining of S-100 is useful in differential diagnosis, although several cases reported positive staining of S-100. Considering the scarce immunohistochemical data on PMTs and the rare cases of positive staining of S-100, we could not confirm the difference between TIO with positive S-100 and TIO with negative S-100. We speculate that several PMTs have a neuroendocrine cause, similar to what Stone et al. reported (35).

Local invasion is commonly identified in PMTs through imaging tests, especially in sinonasal PMTs. Sinonasal PMTs can extend to the cranial fossa, orbital floor, other paranasal sinuses, intracranial tissues, pterygopalatine fossa, infratemporal fossa, and the nasopharynx. Notably, local invasion into surrounding tissues in histopathology is common in PMTMECT (32). TIOs are usually caused by benign tumors, even though they have the features of local invasion. Malignant PMTs were also reported (36). Complete surgery is extremely important in the case of TIO, and a second surgery is required in the case of recurrence. Radiotherapy, tumor embolization, and repeated surgery are preferred in the case of complication or metastasis.

## 5 Limitation

There are some limitations in this study. Firstly, there was not enough detailed information on local invasion, especially in the case series study. Even the number of local invasions might have been underestimated in case reports because not every case had detailed imaging data reported. Secondly, the biochemical results were missing in quite a number of cases. This made the comparison of the real data between sphenoid TIOs and non-sphenoid TIOs difficult. Thirdly, the incidence of local symptoms may be underestimated since it is easily overlooked by physicians.

## 6 Conclusion

TIO is a rare disease caused by tumors located in any part of the body. However, the sphenoid sinus is a rare site for TIO. Functional imaging is crucial to locating the culprit tumor when more than one tumor is found concurrently. Awareness and a timely diagnosis of TIO are crucial for a better outcome.

## Data availability statement

The original contributions presented in the study are included in the article/**Supplementary Material**. Further inquiries can be directed to the corresponding author.

## Ethics statement

The studies involving human participants were reviewed and approved by Tongji Hospital Institutional Review Board. The patients/participants provided written informed consent to participate in this study.

## Author contributions

FW and WH analyzed the data. FW and GY conceived and designed the research. FW, DM, and WX prepared the figures. FW, DM, and JX prepared the Supplementary material. FW drafted the manuscript. GY edited and revised manuscript. All authors contributed to the article and approved the submitted version.
