# Supplementary material for: Sphenoid sinus is a rare site for tumor-induced osteomalacia: A case report and literature review
Source: Front Endocrinol (Lausanne). 2023 Mar 24;14:1116793. doi: 10.3389/fendo.2023.1116793 (PMC10081489; doi:10.3389/fendo.2023.1116793)
Supplement: Supplementary file 1 [file Table_1.docx]

| Supplementary Table 1. The TIO cases occurred in sinonasal sinus (except for sphenoid sinus) in the literature | | | | | | | | |  |
| --- | --- | --- | --- | --- | --- | --- | --- | --- | --- |
|  | Author, year | Duration of disease (m) | Myopathy and osteomalacia | Other clinical symptoms | Serum phosphate (mmol/l) | ALP (U/L) | 1,25–dihydroxy vitamin D (pg/ml) | PTH | FGF23 |
| 1 | Agaimy et al, 2017 [1] | NA | NA | Not in detail | NA | – | – | – | – |
| 2 | Agaimy et al, 2017 [1] | <1 | No | Left eye swelling and loss of vision | normal | – | – | – | – |
| 3 | Agaimy et al, 2017 [1] | 12 | Yes | Epistaxis, anosmia | 0.46 | – | – | – | – |
| 4 | Allevi et al, 2014 [2] | NA | Yes | No | NA | – | 7 | Normal | – |
| 5 | Arnaoutakis et al, 2015 [3] | several | Yes | Nasal airway obstruction, midfacial pressure and pain | NA | 224 | – | – | – |
| 6 | Beech et al, 2007 [4] | 84 | Yes | No | Low | High | – | High | – |
| 7 | Brandwein–Gensler et al, 2012 [5] | many years | Yes | Nose bleed, headache, nasal obstruction | 0.58 | 222 | – | Normal | – |
| 8 | Brociek–Piłczyńska et al, 2022 [6] | 72 | Yes | Nose bleed | 0.64 | 279 | – | 1.51 | 7.93 |
| 9 | Burnand et al, 2012 [7] | 72 | Yes | NA | 0.64 | 293 | – | Unremarkable | 1.57 |
| 10 | Chacko et al, 1981 [8, 9] | 60 | Yes | NA | 0.58 | – | – | – | – |
| 11 | Chanukya et al, 2017 [10] | 24 | Yes | NA | 0.52 | 255 | 4.62 | Normal | 8.74 |
| 12 | Chen et al, 2020 [11] | NA | Yes | NA | 0.58 | 165 | 18 | 2.2 | 1.47 |
| 13 | Chen et al, 2020 [11] | 36 | Yes | NA | 0.68 | 441 | 20 | 0.71 | 3.01 |
| 14 | Chiam et al, 2013 [12] | 18 | Yes | NA | 0.64 | 200 | – | Normal | 1.29 |
| 15 | Cho et al, 2012 [13] | 36 | Yes | Stuffy nose and postnasal dripping | 0.45 | 232 | 22 | 1.36 | – |
| 16 | Clunie et al, 2000 [14] | 60 | Yes | NA | 0.55 | 19KA units | – | – | – |
| 17 | Ding et al, 2018 [15] | 48 | Yes | NA | 0.56 | – | – | – | – |
| 18 | Folpe et al, 2004 [16] | 36 | Yes | NA | NA | – | – | – | – |
| 19 | Fuentealba et al, 2003 [17] | 60 | Yes | NA | 0.45 | 625 | Undetectable | Normal | – |
| 20 | Ghosh et al, 2009 [18] | 48 | Yes | Nasal stuffiness and frontal headache | 0.53 | 1366 | 12.6 | 0.27 | – |
| 21 | González et al, 2017 [19] | 72 | Yes | Nasal obstruction | 0.52 | 340 | – | 1.44 | 1.41 |
| 22 | Gonzalez–Compta et al, 1998 [20] | 24 | Yes | Irrelevant cerebral hemorrhage associated symptoms | NA | – | – | High | NA |
| 23 | Gore et al, 2009 [21] | 96 | Yes | No | 0.23 | 168 | 6 | 1.37 | 2.49 |
| 24 | Gresham et al, 2017 [22] | 36 | Yes | Sinus congestion, decreased sense of smell, and occasional blurry vision | 0.52 | 65.3 | <8 | NA | NA |
| 25 | Hana et al, 2017 [23] | 108 | Yes | Olfactory disturbance | 0.42 | – | 20.5 | NA | 2.4 |
| 26 | Hioco et al, 1981 [9] | – | – | – | – | – | – | – | – |
| 27 | Huang et al, 2019 [24] | 132 | Yes | Epistaxis | 0.33 | 203.7 |  | High | – |
| 28 | Inokuchi et al, 2006 [25] | 4 | Yes | exophthalmos and epiphora | 0.65 | 1823 | 26 | 1.17 | 5.76 |
| 29 | Jamal et al, 2013 [26] | 36 | Yes | NA | 0.44 | 315 | 83.1 | 0.95 | 3.59 |
| 30 | Jefferis et al, 1985 [27] | 24 | Yes | Nasal obstuction | 0.5 | 475 | – | – | – |
| 31 | John et al, 2001 [28] | >72 | Yes | Headache, protusio bulbi, double vision | 0.45 | 313 | 13 | 0.64 |  |
| 32 | John et al, 2019 [29] | 60 | Yes | – | 0.55 | 423 | – | – | 2.48 |
| 33 | Kabtouri et al, 2014 [30] | 36 | Yes | – | 0.35 | 372 | 81 | 0.9 | 1.36 |
| 34 | Kane et al, 2017 [31] | 6 | Yes | Epistaxis | 0.84 | – | – | – | High |
| 35 | Kane et al, 2017 [31] | 12 | Yes | NA | – | – | – | – | High |
| 36 | Kane et al, 2017 [31] | 180 | Yes | NA | 0.77 | – | – | – | High |
| 37 | Kane et al, 2017 [31] | 48 | Yes | NA | 0.32 | – | – | – | High |
| 38 | Kane et al, 2017 [31] | 18 | No | Swelling over left lateral nasal region, upward displacement of left eye | 0.9 | – | – | – | – |
| 39 | Kane et al, 2017 [31] | 36 | Yes | Fullness in right maxillary region | 0.65 | – | – | – | High |
| 40 | Kawai et al, 2001 [32] | 180 | Yes | Nasal bleeding | 0.55 | 211 | 7.3 | 0.38 | – |
| 41 | Kenealy et al, 2008 [33] | NA | Yes | NA | 0.29 | 180 | – | – | 7.89 |
| 42 | Kenealy et al, 2008 [33] | NA | Yes | NA | 0.38 | 120 | – | – | 8.96 |
| 43 | Kominek et al, 2011 [34] | 12 | No | Enlarged nose root | NA | – | – | – | – |
| 44 | Koriyama et al, 2006 [35] | 36 | Yes | NA | 0.55 | 505 | 10.8 | 1.52 | 6.18 |
| 45 | Kurien et al, 2019 [36] | 24 | Yes | NA | 0.42 | 426 | – | 0.95 | 2.86 |
| 46 | Kurien et al, 2019 [36] | 24 | Yes | NA | 0.45 | 105 | – | 0.78 | – |
| 47 | Kurien et al, 2019 [36] | 36 | Yes | NA | 0.58 | 239 | – | 0.27 | 6.64 |
| 48 | Kurien et al, 2019 [36] | 48 | Yes | NA | 0.48 | 332 | – | 1.47 | 1.59 |
| 49 | Kurien et al, 2019 [36] | 48 | No | Epistaxis | – | – | – | – | – |
| 50 | Kurien et al, 2019 [36] | 36 | Yes | NA | 0.61 | 147 | – | 1.15 | 7.64 |
| 51 | Lee et al, 1995 [37] | 36 | Yes | Nasal obstruction | 0.58 | 223 | <5 | Normal | – |
| 52 | Lee et al, 2014 [38] | 62 | Yes | No | 0.45 | 466 | – | Normal | – |
| 53 | Lee et al, 2017 [39] | 42 | Yes | No | 0.55 | 358 | 4.8 | 0.91 | 9.85 |
| 54 | Li et al, 2018 [40] | 12 | Yes | Nasal obstruction, pus snot | 0.17 | 210 | 18 | 2.21 | NA |
| 55 | Massaccesi et al, 2021 [41] | 360 | Yes | NA | NA | – | – | – | – |
| 56 | Mathis et al, 2013 [42] | > 24 | Yes | Epistaxis, intracranial hemorrhage | 0.32 | – | – | – | – |
| 57 | Mok et al, 2016 [43] | 12 | Yes | Epistaxis, anosmia | 0.46 | – | – | – | – |
| 58 | Muniz et al, 2021 [44] | many years | Yes | Epistaxis and nasal obstruction | Low | – | Low | – | – |
| 59 | Nortman et al, 1981 [9] | 48 | Yes | NA | 0.48 | – | – | – | – |
| 60 | Ohashi et al, 1999 [45] | 17 | Yes | Nasal congestion, epistaxis | 0.61 | 392 | 5.9 | 0.87 | – |
| 61 | Okamiya et al, 2015 [46] | 8 | Yes | NA | 0.45 | 738 | 21 | 0.58 | 3.68 |
| 62 | Pal et al, 2019 [47] | Not specific | Not specific | NA | 0.58 | 351 | – | 0.82 | 5.43 |
| 63 | Pal et al, 2019 [47] | Not specific | Not specific | NA | 0.48 | 324 | – | 0.46 | 8.26 |
| 64 | Pal et al, 2019 [47] | Not specific | Not specific | NA | 0.39 | 160 | – | 1.48 | 3.42 |
| 65 | Pal et al, 2019 [47] | Not specific | Not specific | NA | 0.65 | 467 | – | 0.94 | 16.05 |
| 66 | Papierska et al, 2013 [48] | NA | Yes | NA | 0.41 | 137 | – | Normal | 2.48 |
| 67 | Papotti et al, 1988 [49] | 24 | Yes | Nasal bleeding, left exophthalmos | 0.38 | 2430 | Normal | Normal | – |
| 68 | Parshwanath et al, 2010 [50] | 42 | Yes | – | 0.42 | 195 | – | – | – |
| 69 | Pedrazzoli et al, 2010 [51] | 30 | Yes | NA | Low | – | – | – | – |
| 70 | Peterson et al, 2010 [52] | 60 | Yes | NA | Low | High | – | Normal | – |
| 71 | Ray et al, 2015 [53] | 24 | Yes | No | 0.48 | 1756 | – | 0.75 | >5 |
| 72 | Renton et al, 1976 [54] | 36 | Yes | NA | 0.39 | 300KAU/L | – | – | – |
| 73 | Rigante et al, 2019 [55] | NA | Yes | NA | low | – | – | High | High |
| 74 | Sandhu et al, 2000 [56] | 18 | Yes | NA | 0.52 | – | – | – | – |
| 75 | Seshadri et al, 1985 [57] | NA | Yes | NA | 0.32 | – | 9.23 | – | – |
| 76 | Shah et al, 2019 [58] | 12 | Yes | Epistaxis | 0.29 | – | – | – | 4.31 |
| 77 | Shah et al, 2019 [58] | 48 | Yes | No | 0.29 | – | – | – | 5.25 |
| 78 | Shah et al, 2019 [58] | 84 | Yes | Epistaxis, nasal obstruction | 0.61 | – | – | – | 13.49 |
| 79 | Shelekhova et al, 2010 [59] | NA | Yes | NA | – | – | – | – | – |
| 80 | Shelekhova et al, 2010 [59] | NA | Yes | NA | – | – | – | – | – |
| 81 | Sweet et al, 1980 [60] | 12 | Yes | NA | 0.48 | 52 | 10.5 | 0.95 | – |
| 82 | Tang et al, 2020 [61] | 228 | Yes | Nasal obstruction | Not specific | – | – | – | – |
| 83 | Tang et al, 2020 [61] | 24 | Yes | Nasal obstruction | Not specific | – | – | – | – |
| 84 | Tang et al, 2020 [61] | 30 | Yes | No | Not specific | – | – | – | – |
| 85 | Tang et al, 2020 [61] | 60 | Yes | Nasal obstruction | Not specific | – | – | – | – |
| 86 | Tang et al, 2020 [61] | 12 | Yes | Nasal obstruction, purulent nasal discharge | Not specific | – | – | – | – |
| 87 | Tang et al, 2020 [61] | 96 | Yes | Hyposmia | Not specific | – | – | – | – |
| 88 | Tang et al, 2020 [61] | 24 | Yes | Nasal obstruction, purulent nasal discharge | Not specific | – | – | – | – |
| 89 | Tang et al, 2020 [61] | 60 | Yes | Nasal obstruction | Not specific | – | – | – | – |
| 90 | Then et al, 2020 [62] | 12 | Yes | No | 0.52 | 114 | 13 | 2.85 | 2.02 |
| 91 | Villepelet et al, 2018 [63] | NA | Yes | Nasal obstruction | – | – | – | – | – |
| 92 | Xian–Ling et al, 2012 [64] | 24 | Yes | Esotropia in right eye and horizontal diplopia | 0.55 | 358.7 | – | 0.78 | – |
| 93 | Wang et al, 2014 [65] | 48 | Yes | Nasal obstruction | 0.63 | 436 | – | 0.7 | – |
| 94 | Weidner et al, 1985 [66] | 42 | Yes | – | 0.52 | High | 10 | normal |  |
| 95 | Yu et al, 2017 [67] | 12 | Yes | – | 0.38 | 171 | – | 0.54 | 6.76 |
| 96 | Yu et al, 2017 [67] | 72 | Yes | – | 0.41 | 351 | – | 0.48 | 5.26 |
| 97 | Zhu et al, 2021 [68]* | 96 | Likely Yes | Not specific | – | – | – | – | – |
| 98 | Zhu et al, 2021 [68] | 48 | Likely Yes | Not specific | – | – | – | – | – |
| 99 | Zhu et al, 2021 [68] | 12 | Likely Yes | Not specific | – | – | – | – | – |
| 100 | Zhu et al, 2021 [68] | 24 | Likely Yes | Not specific | – | – | – | – | – |
| 101 | Zhu et al, 2021 [68] | 48 | Likely Yes | Not specific | – | – | – | – | – |
| 102 | Zhu et al, 2021 [68] | 36 | Likely Yes | Not specific | – | – | – | – | – |
| 103 | Zhu et al, 2021 [68] | 72 | Likely Yes | Not specific | – | – | – | – | – |
| 104 | Zhu et al, 2021 [68] | 18 | Likely Yes | Not specific | – | – | – | – | – |
| 105 | Zhu et al, 2021 [68] | 36 | Likely Yes | Not specific | – | – | – | – | – |
| 106 | Zhu et al, 2021 [68] | 18 | Likely Yes | Not specific | – | – | – | – | – |
| 107 | Zhu et al, 2021 [68] | 48 | Likely Yes | Not specific | – | – | – | – | – |
| 108 | Zhu et al, 2021 [68] | 6 | Likely Yes | Not specific | – | – | – | – | – |
| 109 | Zhu et al, 2021 [68] | 24 | Likely Yes | Not specific | – | – | – | – | – |
| 110 | Zhu et al, 2021 [68] | 72 | Likely Yes | Not specific | – | – | – | – | – |
| 111 | Zhu et al, 2021 [68] | 60 | Likely Yes | Not specific | – | – | – | – | – |
| 112 | Zhu et al, 2021 [68] | 60 | Likely Yes | Not specific | – | – | – | – | – |
| 113 | Zhu et al, 2021 [68] | 36 | Likely Yes | Not specific | – | – | – | – | – |
| 114 | Zhu et al, 2021 [68] | 96 | Likely Yes | Not specific | – | – | – | – | – |
| 115 | Zhu et al, 2021 [68] | 60 | Likely Yes | Not specific | – | – | – | – | – |
| 116 | Zhu et al, 2021 [68] | 18 | Likely Yes | Not specific | – | – | – | – | – |
| 117 | Zhu et al, 2021 [68] | 6 | Likely Yes | Not specific | – | – | – | – | – |
| 118 | Zhu et al, 2021 [68] | 36 | Likely Yes | Not specific | – | – | – | – | – |
| 119 | Zhu et al, 2021 [68] | 24 | Likely Yes | Not specific | – | – | – | – | – |
| 120 | Zhu et al, 2021 [68] | 36 | Likely Yes | Not specific | – | – | – | – | – |
| 121 | Zhu et al, 2021 [68] | 60 | Likely Yes | Not specific | – | – | – | – | – |
| 122 | Zhu et al, 2021 [68] | 24 | Likely Yes | Not specific | – | – | – | – | – |
| 123 | Zhu et al, 2021 [68] | 108 | Likely Yes | Not specific | – | – | – | – | – |
| 124 | Zhu et al, 2021 [68] | 36 | Likely Yes | Not specific | – | – | – | – | – |
| 125 | Zhu et al, 2021 [68] | 72 | Likely Yes | Not specific | – | – | – | – | – |
| 126 | Zhu et al, 2021 [68] | 12 | Likely Yes | Not specific | – | – | – | – | – |
| 127 | Zhu et al, 2021 [68] | 12 | Likely Yes | Not specific | – | – | – | – | – |
| 128 | Zhu et al, 2021 [68] | 24 | Likely Yes | Not specific | – | – | – | – | – |
| 129 | Zhu et al, 2021 [68] | 72 | Likely Yes | Not specific | – | – | – | – | – |
| 130 | Zhu et al, 2021 [68] | 72 | Likely Yes | Not specific | – | – | – | – | – |
| 131 | Zhu et al, 2021 [68] | 156 | Likely Yes | Not specific | – | – | – | – | – |
| 132 | Zhu et al, 2021 [68] | 60 | Likely Yes | Not specific | – | – | – | – | – |
| 133 | Zhu et al, 2021 [68] | 72 | Likely Yes | Not specific | – | – | – | – | – |
| 134 | Zhu et al, 2021 [68] | 36 | Likely Yes | Not specific | – | – | – | – | – |
| 135 | Zhu et al, 2021 [68] | 36 | Likely Yes | Not specific | – | – | – | – | – |
| 136 | Zuo et al, 2017 [69] | 36 | Yes | NA | 0.24 | 96 | – | 1.09 | – |

| Supplementary Table 1. The TIO cases occurred in sinonasal sinus (except for sphenoid sinus) in the literature | | | | | | | | |
| --- | --- | --- | --- | --- | --- | --- | --- | --- |
|  | Author, year | Sex/Age | Tumor site | Histological diagnosis | Treatment | Outcome and recurrence | Tumor invasion | Immunohistochemistry |
| 1 | Agaimy et al, 2017 [1] | M/52 | Left ethmoid | Hemangiopericytoma + cellular glomoid | Surgery | Cured, NED (7m) | NA | Positive for SSTR2A, SATB2, ERG, CD56. Negative for S–100, CD34, DOG1, STAT6 and beta–catenin |
| 2 | Agaimy et al, 2017 [1] | F/45 | Left frontal sinus | PMT | Surgery | Cured, NED (NA) | Very likely | Positive for SSTR2A, SATB2, ERG, CD56. Negative for S–100, CD34, DOG1, STAT6 and beta–catenin |
| 3 | Agaimy et al, 2017 [1] | M/48 | Nasal cavity | PMT | Surgery | Cured, NED (12m) | Local invasion | Positive for SSTR2A, SATB2, ERG, CD56. Negative for S–100, CD34, DOG1 and beta–catenin |
| 4 | Allevi et al, 2014 [2] | F | Right maxillary sinus | PMT | Resurgery | Recurrence at 120m after the first surgery, cured and NED (48m) after the second surgery | Unlikely | NA |
| 5 | Arnaoutakis et al, 2015 [3] | F/50 | Right frontal recess, middle meatus, anterior ethmoid, cribriform plate of ethmoid bone | PMT | Surgery | Cured | Local invasion | Negative for AE1/AE3, NSE, neurofilament, CgA, Syn, S–100, desmin, SMA and CD34 |
| 6 | Beech et al, 2007 [4] | M/42 | Right ethmoid sinus | Hemangiopericytoma | Surgery | Cured | No | NA |
| 7 | Brandwein–Gensler et al, 2012 [5] | F/66 | Right maxillary sinus, ethmoids, nasal cavity with extension to cribiform plate | Glomangiopericytoma | Surgery | Cured, NED (25m) | Local invasion | NA |
| 8 | Brociek–Piłczyńska et al, 2022 [6] | F/42 | Left ethmoid and frontal sinus, invading the wall of the left orbit with possible intracranial tumor spread | Glomangiopericytoma | Surgery | NED (16m) with tumor residual | Local invasion | Positive for podoplanin and cyclin–D. Negative for CD34, CK, AE1/3, EMA, S–100, desmin, SMA, HMB45 |
| 9 | Burnand et al, 2012 [7] | M/54 | Left nasal cavity, obstructing the maxillary sinus, middle turbinate and ethmoid | Hemangiopericytoma | Surgery | Cured, NED (6m) | Local invasion | NA |
| 10 | Chacko et al, 1981 [8, 9] | F/56 | Ethmoid | Hemangiopericytoma | Incomplete resection and radiotherapy | Died of epistaxis | NA | Could not find |
| 11 | Chanukya et al, 2017 [10] | M/31 | Left nasal cavity and ipsilateral maxillary sinus | Hemangiopericytoma | Surgery | Cured, NED (3m) | Local invasion | NA |
| 12 | Chen et al, 2020 [11] | F/59 | Right middle turbinate | PMT | Surgery | Cured, NED (1m) | No | NA |
| 13 | Chen et al, 2020 [11] | M/61 | Right frontal sinus invading bone and abutment of the dura | PMT | Surgery | Cured, NED (13m) | Local invasion | NA |
| 14 | Chiam et al, 2013 [12] | M/55 | Right nasal cavity | PMT | Surgery | Cured, NED (3m) | No | NA |
| 15 | Cho et al, 2012 [13] | F/47 | Left nasal cavity and posterior ethmoid sinus | Hemangiopericytoma | Surgery | Cured, NED (1m) | Local invasion | NA |
| 16 | Clunie et al, 2000 [14] | F/60 | Ethmoid sinus | Hemangiopericytoma | Surgery | Recurred 48 m after surgery and died of colon cancer metastasis | No | NA |
| 17 | Ding et al, 2018 [15] | F/66 | Right nasal cavity | NA | Surgery | Cured, NED (NA) | No | NA |
| 18 | Folpe et al, 2004 [16] | M/46 | Ethmoid sinus | Hemangiopericytoma–like tumor | Resurgery | Recurred 1 year after first surgery, NED (144m) after second surgery | No | NA |
| 19 | Fuentealba et al, 2003 [17] | M/50 | Nasal cavity and left maxillary sinus | Hemangiopericytoma | Resurgery, embolization of the tumor, and adjuvant radiotherapy | Recurrence at 3m after first surgery and died of bronchopneumonia | Local invasion | NA |
| 20 | Ghosh et al, 2009 [18] | M/48 | Nasopharyngeal tumor with an extension along the left inferior turbinate | Hemangiopericytoma | Surgery | Cured, NED (3m) | Local invasion | NA |
| 21 | González et al, 2017 [19] | M/42 | Left nasal fossa with extension to frontal sinus | PMT | Surgery | Cured, NED (6m) | Local invasion | NA |
| 22 | Gonzalez–Compta et al, 1998 [20] | F/69 | Right ethmoid, frontal sinus, maxillary sinus, cribiform plate, and anterior cranial fossa | PMT | Medical treatment | Died of cerebral hemorrhage before surgery | Local invasion | NA |
| 23 | Gore et al, 2009 [21] | F/52 | Nasal cavity | PMT | Surgery | Cured, NED (27m) | No | NA |
| 24 | Gresham et al, 2017 [22] | M/42 | Ethmoid sinus extending through the cribriform | Glomangioma | Surgery | Cured, NED (12m) | No | Positive for CD31 and SMA. Negative for CD34, S100 and pancytokeratin |
| 25 | Hana et al, 2017 [23] | M/38 | Bilateral ethmoid sinus extruding into frontal skull base | PMT | Surgery | Cured, NED (25m) | Local invasion | NA |
| 26 | Hioco et al, 1981 [9] | M/69 | Nose | Angiofibroma | NA | Recurrence, unknown outcome | Could not find | Could not find |
| 27 | Huang et al, 2019 [24] | F/42 | Nasal tumor invading frontal sinus and ethmoid sinus | Hemangiopericytoma | Surgery | Cured, NED (6m) | Local invasion | Positive for CD34, Bcl–2 and CD56. Negative for CK, EMA, S100, HMB45, Melan–A, desmin, Syn, CgA, LCA, CD3, CD20, CD79a, SMA, Myogenin, MyoD1, TiA–1, CD43, EBER, GFAP |
| 28 | Inokuchi et al, 2006 [25] | F/24 | Right nasal cavity, maxillary sinus, anterior ethmoid sinus and frontal sinus, destroyed the medial wall of the right orbit | PMT | Embolism and surgery | Cured, NED (12m) | Local invasion | NA |
| 29 | Jamal et al, 2013 [26] | M/55 | Left posterior nasal cavity | PMT | Radiation and resection | Cured, NED (4m) | No | Positive for Bcl–2. Negative for SMA, desmin, CD34, S–100, AE1/AE3 |
| 30 | Jefferis et al, 1985 [27] | F/27 | Maxillary sinus and invading orbital floor, anterior antral wall and pterygopalatine fossa | Odontogenic tumor | Surgery | Cured, NED (6m) | Local invasion | NA |
| 31 | John et al, 2001 [28] | F/54 | Right frontal, nasal ethmoidal, right maxillary sinus, right orbita, periorbital bone | Malignant schwannoma | Surgery and radiation therapy | Controlled, phosphate is still low at 24m post operation but normal at 30m and 36m post operation | Local invasion | NA |
| 32 | John et al, 2019 [29] | M/50 | Ethmoid sinus | NA | Oral medicine | NA | NA | NA |
| 33 | Kabtouri et al, 2014 [30] | F/42 | Nasal cavity and ethmoid | Hemangiopericytoma | Surgery | Cured, NED (36m) | Local invasion | NA |
| 34 | Kane et al, 2017 [31] | M/62 | Left nasal cavity, extending to ethmoid and frontal sinuses, invading lamina papyracea | PMT | Surgery followed by radiotherapy | Cured, NED (7m) | Local invasion | Positive for Bcl–2. Negative for SMA, CD34 and S–100 |
| 35 | Kane et al, 2017 [31] | F/56 | Left nasal and spheno–ethmoidal mass | PMT | NA | NA | Local invasion | Negative for SMA |
| 36 | Kane et al, 2017 [31] | M/52 | Left nasal cavity | PMT | Surgery | Cured, NED (15m) | No | Positive for vimentin. Negative for SMA. |
| 37 | Kane et al, 2017 [31] | M/39 | Right basi–fontal region, right ethmoidal sinus | PMT | Resurgery | Cured, NED (10m) | Local invasion | Negative for EMA, SMA, CD34 and Bcl–2 |
| 38 | Kane et al, 2017 [31] | M/34 | Left nasal cavity, maxillary sinus, anterior wall of left middle cranial fossa, the orbit roof and left optic nerve | PMT | Surgery followed by radiotherapy | Cured, NED (6m) | Local invasion | Negative for SMA. Positive for CD34 and Bcl–2. Focal positive for S–100 |
| 39 | Kane et al, 2017 [31] | F/35 | Right upper alveolus, anterior wall of maxilla, with associated soft tissue component | PMT | Surgery | Cured | No | Positive for vimentin. Focal positive for CD68. Negative for CD34, S–100 and ER |
| 40 | Kawai et al, 2001 [32] | F/53 | Right ethmoid and nasal cavity | PMT | Surgery | Cured | Local invasion | NA |
| 41 | Kenealy et al, 2008 [33] | F/79 | Ethmoid sinus | PMT | Surgery | Cured | NA | NA |
| 42 | Kenealy et al, 2008 [33] | F/40 | Ethmoid sinus | Hemangiopericytoma | Surgery | Cured | NA | NA |
| 43 | Kominek et al, 2011 [34] | M/53 | Bilateral ethmoids, frontal sinus, and nasal cavity | PMT | Surgery | Cured, NED (24m) | Local invasion | Focally positive for SMA, strong positive for vimentin. Negative for CD 34, CD 99, S–100, AE1 and AE3 |
| 44 | Koriyama et al, 2006 [35] | F/41 | Maxillary sinus | PMT | Surgery | Cured, NED (588m) | No | Positive for FGF23, vimentin, SSTR2A |
| 45 | Kurien et al, 2019 [36] | F/39 | Right nasal cavity and middle turbinate | PMT | Surgery | Cured, NED (13m) | Local invasion | NA |
| 46 | Kurien et al, 2019 [36] | F/36 | Left anterior ethmoid and posterior ethmoid | PMT | Surgery | Cured, NED (58m) | Local invasion | Positive for SMA |
| 47 | Kurien et al, 2019 [36] | M/51 | Middle turbinate and septum | PMT | Surgery | Cured, NED (24m) | Local invasion | NA |
| 48 | Kurien et al, 2019 [36] | M/44 | Middle turbinate and frontal recess | PMT | Surgery | Cured, NED (6m) | Local invasion | Positive for vimentin, Bcl–2. Negative for EMA |
| 49 | Kurien et al, 2019 [36] | M/39 | Middle turbinate, cribriform plate, septum | PMT | Surgery | Cured, NED (3m) | Local invasion | Positive for vimentin, TLE–1 and CD34. Negative for CK, SMA, EMA and S–100 |
| 50 | Kurien et al, 2019 [36] | F/37 | Anterior ethmoid, cribriform plate, nasal cavity, lamina, extraconal involvement of the orbit, intracranial extradural | Malignant PMT | Surgery and radiotherapy | Normal serum phosphate | Local invasion | NA |
| 51 | Lee et al, 1995 [37] | F/66 | Nasal cavity and invading the right orbit | Hemagiopericytoma | Surgery | Cured, NED (10m) | Local invasion | NA |
| 52 | Lee et al, 2014 [38] | F/60 | Maxillary cavity | Glomangiopericytoma | Resurgery | Cured, NED (10m) | No | Positive for SMA. Negative for CD31 and CD34. |
| 53 | Lee et al, 2017 [39] | M/52 | Left ethmoid sinus | PMT | Surgery and radiation therapy | Cured, NED (NA) | No |  |
| 54 | Li et al, 2018 [40] | F/40 | Left nasal cavity | Hemangiopericytoma | Resurgery and medication | Live with disease after first surgery and nasal obstruction relapsed six years later. NED (6m) after second surgery | No | Positive for cyclin D1, STAT–6, SMA and FGF23 |
| 55 | Massaccesi et al, 2021 [41] | M/52 | Left frontal sinus | ossifying fibromyxoid tumor | Four surgeries and radiation therapy | Frequent recurrence after the first three surgery and NED (60m) after the fourth surgery and radiation | No | NA |
| 56 | Mathis et al, 2013 [42] | M/32 | Anterior cranial  fossa, ethmoid  sinus, nasal cavity | Haemangiopericytoma, revised as PMT | Multiple surgeries | Recurrence without specific information | Local invasion | Positive for vimentin and SMA, negative for CD34, EMA, GFAP, Syn, calponin, collagen Type IV and keratin |
| 57 | Mok et al, 2016 [43] | M/48 | Right nasal cavity, right maxillary sinus, bilateral ethmoid, nasopharynx | PMT | Surgery | Cured, NED (12m) | Local invasion | Negative for SMA, CD34, desmin, S–100 and AE1/AE3. |
| 58 | Muniz et al, 2021 [44] | F/32 | Right nasal cavity and ethmoid sinus | Glomangioma | surgery | Cured, NED (24m) | No | Positive for CD34 and SMA |
| 59 | Nortman et al, 1981 [9] | F/49 | Nose | Mesenquimal tumor | Resurgery | NA | NA | NA |
| 60 | Ohashi et al, 1999 [45] | M/43 | Left maxillary sinus | Hemangiopericytoma–like tumor | Surgery | Cured, NED (0.5m) | No | NA |
| 61 | Okamiya et al, 2015 [46] | F/35 | Left anterior ethmoid sinus | PMT | Surgery | Cured, NED (36m) | No | Positive for FGF23. Focal positive SMA. Negative for desmin, S–100, keratin and CD34 |
| 62 | Pal et al, 2019 [47] | F/52 | Right nasal cavity | Arteriovenous hemangioma | Surgery | Cured, NED (NA) | No | NA |
| 63 | Pal et al, 2019 [47] | F/36 | Right maxillary sinus | PMT | Surgery | Cured, NED (NA) | No | NA |
| 64 | Pal et al, 2019 [47] | M/58 | Left nasal cavity | Hemangiopericytoma | Surgery | Cured, NED (NA) | No | NA |
| 65 | Pal et al, 2019 [47] | F/36 | Left nasal cavity | Hemangiopericytoma | Surgery | Cured, NED (NA) | No | NA |
| 66 | Papierska et al, 2013 [48] | 40 | Right maxillary sinus | Glomangiopericytoma | Surgery | Cured, NED (NA) | No | NA |
| 67 | Papotti et al, 1988 [49] | F/38 | Left maxillary sinus, ethmoid, and nasal cavity with orbital floor invasion | Hemangiopericytoma–like tumor, revised as PMT | Partial surgery and adjuvant radiotherapy | Alleviated, AD (21m) | Local invasion | Positive for vimentin. Negative for factor VIII and S–100 |
| 68 | Parshwanath et al, 2010 [50] | F/42 | Left nasal cavity and ethmoid sinus | PMT | Surgery | Cured, NED (3m) | Local invasion | NA |
| 69 | Pedrazzoli et al, 2010 [51] | F/37 | Maxillary sinus | PMT | Surgery | Cured, NED (84m) | No | Positive for vimentin, focal positive for SMA, CD68 and S100 |
| 70 | Peterson et al, 2010 [52] | F/33 | Maxillary sinus, buccal space and tibia | PMT | One surgery of tibia and one surgery in maxillary sinus and medical therapy | Not cured | Local invasion | NA |
| 71 | Ray et al, 2015 [53] | M/35 | Left nasal cavity, nasal septum and lateral wall of nasal cavity | Hemangiopericytoma–like tumor | Surgery | Cured, NED (0.5m) | No | NA |
| 72 | Renton et al, 1976 [54] | F/51 | Ethmoid | Hemagiopericytoma | Partial resection and medical therapy | Not cured | Local invasion | NA |
| 73 | Rigante et al, 2019 [55] | M/52 | Frontal sinus | Ossifying fibromyxoid tumor | Surgery | Cured, NED (24m) | No | Positive for vimentin, focal and weak positive for S100. Negative for NSE, CD10, GFAP, CD31, CD34, LCA, ASMA, SMMHC, desmin, CD99, Bcl2, and AE1/AE3 |
| 74 | Sandhu et al, 2000 [56] | M/46 | Right ethmoid sinus invading brain parenchyma and dura | Hemangiopericytoma | Surgical excision and adjuvant radiotherapy | Cured, NED (22m) | No | Positive for NSE, vimentin and SMA. Negative for EMA, CK, S–100, chromogranin, CD57 glial fibrillary protein and desmin |
| 75 | Seshadri et al, 1985 [57] | F/40 | Ethmoid | Hemangiopericytoma | Surgery | Cured | No | NA |
| 76 | Shah et al, 2019 [58] | M/60 | Left ethmoid sinus | PMT | Multiple surgery and radiation therapy | Cured, NED (36m) | NA | NA |
| 77 | Shah et al, 2019 [58] | M/39 | Right frontal and ethmoid sinus | PMT | Resurgery | Cured, NED (29m) | NA | NA |
| 78 | Shah et al, 2019 [58] | M/36 | Right nasal cavity | PMT | Surgery | Cured, NED (2m) | NA | NA |
| 79 | Shelekhova et al, 2010 [59] | F/70 | Sinonasal cavity (specific information is not available) | PMT | Surgery | NA | No | NA |
| 80 | Shelekhova et al, 2010 [59] | M/53 | Frontal sinus | PMT | Surgery | NA | No | NA |
| 81 | Sweet et al, 1980 [60] | F/25 | Left middle turbinate (nasal cavity) | Hemangiopericytoma | Resurgery | Cured, NED (NA) | No | NA |
| 82 | Tang et al, 2020 [61] | M/59 | Left nasal cavity and ethmoid sinus | PMT | Surgery | Cured, NED (12.5m) | Local invasion | Negative for desmin and S–100 |
| 83 | Tang et al, 2020 [61] | F/56 | Right nasal cavity and ethmoid sinus, nasal septum and the contralateral nasal cavity | PMT | Surgery | Cured, NED (10.5m) | Local invasion | Negative for desmin and S–100 |
| 84 | Tang et al, 2020 [61] | M/42 | Right nasal cavity and ethmoid sinus | PMT | Surgery | Cured, NED (9.5m) | Local invasion | Negative for desmin and S–100 |
| 85 | Tang et al, 2020 [61] | F/43 | Left frontal sinus and ethmoid sinus with extension to the contralateral frontal sinus and anterior ethmoid sinus | PMT | Surgery | Cured, NED (14.5m) | Local invasion | Negative for desmin and S–100 |
| 86 | Tang et al, 2020 [61] | F/54 | Right nasal cavity and ethmoid sinus | PMT | Surgery | Cured, NED (24m) | Local invasion | Negative for desmin and S–100 |
| 87 | Tang et al, 2020 [61] | M/42 | Right nasal cavity and ethmoid sinus, intracranial invasion | PMT | Surgery | Recurrence, died of brain hernia | Local invasion | Negative for desmin and S–100 |
| 88 | Tang et al, 2020 [61] | F36 | Left nasal cavity and ethmoid sinus | PMT | Surgery | Cured, NED (54m) | Local invasion | Negative for desmin and S–100 |
| 89 | Tang et al, 2020 [61] | M/51 | Left nasal cavity and ethmoid sinus | PMT | Surgery | Cured, NED (60m) | Local invasion | Negative for desmin and S–100 |
| 90 | Then et al, 2020 [62] | F/50 | Ethmoid sinus | PMT | Surgery | Cured, NED (30m) | No | Positive for SATB2 and NSE. Negative for CD3, CD20, CD45, GFAP, CD31, S100, STAT6, actin, HMB45, CD99, EMA, TLE, Syn, chromogranin, CD34, CD68, KL1, desmin, and ceratin |
| 91 | Villepelet et al, 2018 [63] | F/41 | Right nasal cavity and ethmoid sinus, frontal sinus. | PMT | surgery | Cured, NED (9m) | Local invasion | NA |
| 92 | Xian–Ling et al, 2012 [64] | F/42 | Left ethmoid and nasal cavity | PMT | Surgery | Cured, NED (NA) | Local invasion | NA |
| 93 | Wang et al, 2014 [65] | M/46 | Nasal cavity, left ethmoid sinus and the floor of maxillary sinus | Glomus tumor | Surgery | Cured, NED (1m) | Local invasion | NA |
| 94 | Weidner et al, 1985 [66] | F/39 | Right maxillary sinus with infratemporal fossa invasion | PMT | Resurgery | Cured, No recurrence at 5 m after second surgery | Local invasion | NA |
| 95 | Yu et al, 2017 [67] | F/38 | Left nasal cavity and ethmoid sinus | PMT | Surgery | Cured, NED (0.5m) | Local invasion | NA |
| 96 | Yu et al, 2017 [67] | M/50 | Left nasal cavity | PMT | Surgery | Cured, NED (1m) | No | NA |
| 97 | Zhu et al, 2021 [68]* | M/36 | Ethmoid sinus, frontal sinus | PMT | Surgery | Cured, NED (43m) | Local invasion | NA |
| 98 | Zhu et al, 2021 [68] | M/36 | Ethmoid sinus | PMT | Surgery | Cured, NED (44m) | No | NA |
| 99 | Zhu et al, 2021 [68] | M/37 | Nasal cavity | PMT | Surgery | Cured, NED (39m) | No | NA |
| 100 | Zhu et al, 2021 [68] | M/39 | Ethmoid sinus | PMT | Surgery | Cured, NED (45m) | No | NA |
| 101 | Zhu et al, 2021 [68] | F/60 | Nasal cavity | PMT | Surgery | Cured, NED (48m) | No | NA |
| 102 | Zhu et al, 2021 [68] | F/56 | Ethmoid Sinus, frontal Sinus | PMT | Surgery | Cured, NED (42m) | Local invasion | NA |
| 103 | Zhu et al, 2021 [68] | F/50 | Ethmoid sinus | PMT | Surgery | Cured, NED (28m) | No | NA |
| 104 | Zhu et al, 2021 [68] | M/35 | Ethmoid sinus | PMT | Surgery | Cured, NED (59m) | No | NA |
| 105 | Zhu et al, 2021 [68] | M/39 | Ethmoid sinus | PMT | Surgery | Cured, NED (63m) | No | NA |
| 106 | Zhu et al, 2021 [68] | M/37 | Frontal sinus | PMT | Surgery | Cured, NED (60m) | No | NA |
| 107 | Zhu et al, 2021 [68] | M/61 | Ethmoid sinus, nasal cavity | PMT | Surgery | Cured, NED (13m) | Local invasion | NA |
| 108 | Zhu et al, 2021 [68] | M/29 | Ethmoid sinus | PMT | Surgery | Cured, NED (17m) | No | NA |
| 109 | Zhu et al, 2021 [68] | M/50 | Ethmoid sinus | PMT | Surgery | Cured, NED (8m) | No | NA |
| 110 | Zhu et al, 2021 [68] | M/19 | Ethmoid sinus | PMT | Surgery | Cured, NED (29m) | No | NA |
| 111 | Zhu et al, 2021 [68] | M/50 | Nasal cavity | PMT | Surgery | Cured, NED (69m) | No | NA |
| 112 | Zhu et al, 2021 [68] | M/40 | Ethmoid sinus | PMT | Surgery | Cured, NED (77m) | No | NA |
| 113 | Zhu et al, 2021 [68] | F/40 | Ethmoid sinus | PMT | Surgery | Cured, NED (5m) | No | NA |
| 114 | Zhu et al, 2021 [68] | F/57 | Ethmoid sinus | PMT | Surgery | Cured, NED (24m) | No | NA |
| 115 | Zhu et al, 2021 [68] | F/52 | Nasal cavity | PMT | Surgery | Cured, NED (30m) | No | NA |
| 116 | Zhu et al, 2021 [68] | F/40 | Ethmoid sinus | PMT | Surgery | Cured, NED (8m) | No | NA |
| 117 | Zhu et al, 2021 [68] | F/39 | Nasal cavity | PMT | Surgery | Cured, NED (77m) | No | NA |
| 118 | Zhu et al, 2021 [68] | F/52 | Ethmoid sinus | PMT | Surgery | Cured, NED (33m) | No | NA |
| 119 | Zhu et al, 2021 [68] | F/36 | Ethmoid sinus | PMT | Surgery | Cured, NED (81m) | No | NA |
| 120 | Zhu et al, 2021 [68] | F/57 | Ethmoid sinus | PMT | Surgery | Cured, NED (18m) | No | NA |
| 121 | Zhu et al, 2021 [68] | F/54 | Ethmoid sinus | PMT | Surgery | Cured, NED (87m) | No | NA |
| 122 | Zhu et al, 2021 [68] | F/43 | Ethmoid sinus | PMT | Surgery | Cured, NED (1m) | No | NA |
| 123 | Zhu et al, 2021 [68] | F/45 | Ethmoid sinus | PMT | Surgery | Cured, NED (144m) | No | NA |
| 124 | Zhu et al, 2021 [68] | F/39 | Ethmoid Sinus, nasal Cavity | PMT | Surgery | Cured, NED (68m) | Local invasion | NA |
| 125 | Zhu et al, 2021 [68] | M/32 | Ethmoid sinus | PMT | Surgery | Cured, NED (8m) | No | NA |
| 126 | Zhu et al, 2021 [68] | M/37 | Ethmoid sinus | PMT | Surgery | Cured, NED (6m) | No | NA |
| 127 | Zhu et al, 2021 [68] | M/44 | Ethmoid sinus, nasal cavity | PMT | Surgery | Cured, NED (6m) | Local invasion | NA |
| 128 | Zhu et al, 2021 [68] | F/45 | Ethmoid sinus, nasal cavity | PMT | Surgery | Cured, NED (5m) | Local invasion | NA |
| 129 | Zhu et al, 2021 [68] | M/27 | Maxillary sinus | Odontogenic fibroma | Surgery | Cured, NED (36m) | No | NA |
| 130 | Zhu et al, 2021 [68] | M/18 | Nasal cavity | Hemangiofibroma | Surgery | Cured, NED (6m) | No | NA |
| 131 | Zhu et al, 2021 [68] | F/36 | Ethmoid sinus | PMT | Surgery | Cured, NED (60m) | No | NA |
| 132 | Zhu et al, 2021 [68] | F/44 | Left ethmoid sinus, right mandible | PMT | Resurgery | Cured, NED (5m) after second surgery | Local invasion | NA |
| 133 | Zhu et al, 2021 [68] | F/49 | Ethmoid sinus; nasal cavity | PMT | Resurgery | Cured, Recurred at 8m after first surgery, NED (1m) after second surgery | Local invasion | NA |
| 134 | Zhu et al, 2021 [68] | F/57 | Nasal cavity, ethmoid sinus | PMT | Resurgery | Not cured after first surgery, NED (63m) after second surgery | Local invasion | NA |
| 135 | Zhu et al, 2021 [68] | M/24 | Ethmoid sinus, frontal sinus | PMT | Resurgery | Not cured after first surgery, NED (137m) after second surgery | Local invasion | NA |
| 136 | Zuo et al, 2017 [69] | M/NA | Left nasal cavity and ethmoid, frontal, and maxillary sinuses | PMT | surgery | Cured, NED(NA) | Local invasion | NA |
|  | | | | | | | | |

En dash indicates that the value is not available.

AD: alive with disease; AE1/AE3: pan cytokeratin; ASMA: Human Anti–smooth muscle antibody; Bcl–2: B–cell lymphoma 2; CD: cluster of differentiation; CgA: chromogranin A; CK: cytokeratin; Could not find: The original article could not find. The basic information was obtained from other researcher’s article; EMA: epithelial membrane antigen; EBER: EBV–encoded RNA; ERG: E26 transformation specific transcription factor; FGF23: fibroblast growth factor 23; Fli–1: Friend leukemia integration–1; GFAP: glial fibrillary acidic protein; HMB45: human melanoma black 45; LCA: leukocyte common antigen, CD45; NA: not available, usually not mentioned in the original article; Melan–A: melanoma antigen recognized by T cell; MyoD1: myogenic differentiation 1; NED: no evidence of disease; NSE: neuron specific enolase; PCK: pan–cytokeratin; PR: progesterone receptor; SATB: special AT rich sequence binding protein; SMA: smooth muscle actin; SMMHC: smooth muscle myosin heavy chain; SOX10: SRY–Box transcription factor 10; SSTR2A: somatostatin receptor 2A; STAT6: signal transducer and activator of transcription 6; Syn: synaptophysin; TiA–1: cytotoxic granule associated RNA binding protein 1; TLE1: transducer–like enhancer split 1;

*: The information comes from a case series without detailed clinical features of each single patient, so the information in "tumor invasion" column may not accurate. Besides, it is likely that Zhu enrolled the cases reported by Jiang [70] although we didn’t get a reply from Zhu, so we ruled out the possible duplicate cases.

References

1. Agaimy, A., M. Michal, S. Chiosea, F. Petersson, L. Hadravsky, G. Kristiansen, R.E. Horch, J. Schmolders, A. Hartmann, F. Haller, and M. Michal, Phosphaturic Mesenchymal Tumors: Clinicopathologic, Immunohistochemical and Molecular Analysis of 22 Cases Expanding their Morphologic and Immunophenotypic Spectrum*.* Am J Surg Pathol, 2017. 41(10): p. 1371–1380. <https://doi.org/10.1097/PAS.0000000000000890>.

2. Allevi, F., D. Rabbiosi, M. Mandala, and G. Colletti, Mesenchymal phosphaturic tumour: early detection of recurrence*.* BMJ Case Rep, 2014. 2014. [https://doi.org/10.1136/bcr–2013–202827](https://doi.org/10.1136/bcr-2013-202827).

3. Arnaoutakis, D. and I. Naseri, Sinonasal Phosphaturic Mesenchymal Tumor: A Rare and Misinterpreted Entity*.* J Neurol Surg Rep, 2015. 76(2): p. e233–8. [https://doi.org/10.1055/s–0035–1562852](https://doi.org/10.1055/s-0035-1562852).

4. Beech, T.J., A. Rokade, N. Gittoes, and A.P. Johnson, A haemangiopericytoma of the ethmoid sinus causing oncogenic osteomalacia: a case report and review of the literature*.* Int J Oral Maxillofac Surg, 2007. 36(10): p. 956–8. <https://doi.org/10.1016/j.ijom.2007.03.005>.

5. Brandwein–Gensler, M. and G.P. Siegal, Striking pathology gold: a singular experience with daily reverberations: sinonasal hemangiopericytoma (glomangiopericytoma) and oncogenic osteomalacia*.* Head Neck Pathol, 2012. 6(1): p. 64–74. [https://doi.org/10.1007/s12105–012–0337–8](https://doi.org/10.1007/s12105-012-0337-8).

6. Brociek–Pilczynska, A., D. Brodowska–Kania, K. Szczygielski, M. Lorent, G. Zielinski, P. Kowalewski, and D. Jurkiewicz, A rare combination of tumor–induced osteomalacia caused by sinonasal glomangiopericytoma and coexisting parathyroid adenoma: case report and literature review*.* BMC Endocr Disord, 2022. 22(1): p. 31. [https://doi.org/10.1186/s12902–022–00934–7](https://doi.org/10.1186/s12902-022-00934-7).

7. Burnand, H., A. Samuels, I. Hagan, N. Sawant, and J. Mutimer, Bilateral subtrochanteric fractures in tumour–induced osteomalacia caused by a nasal haemangiopericytoma*.* Hip Int, 2012. 22(2): p. 227–9. <https://doi.org/10.5301/HIP.2012.9235>.

8. Chacko, V. and B. Joesph, Osteomalacia associated with haemangiopericytoma*.* J Indian Med Assoc, 1981. 76(9): p. 173–5

9. Ryan, E.A. and E. Reiss, Oncogenous osteomalacia. Review of the world literature of 42 cases and report of two new cases*.* Am J Med, 1984. 77(3): p. 501–12. [https://doi.org/10.1016/0002–9343(84)90112–8](https://doi.org/10.1016/0002-9343(84)90112-8).

10. Chanukya, G.V., M. Mengade, J. Goud, I.S. Rao, and A. Jain, Tumor–induced Osteomalacia: A Sherlock Holmes Approach to Diagnosis and Management*.* Ann Maxillofac Surg, 2017. 7(1): p. 143–147. [https://doi.org/10.4103/ams.ams–123–16](https://doi.org/10.4103/ams.ams_123_16).

11. Chen, D.W., G.A. Clines, M.T. Collins, L. Douyon, and P.U. Choksi, A rare cause of atraumatic fractures: case series of four patients with tumor–induced osteomalacia*.* Clin Diabetes Endocrinol, 2020. 6: p. 12. [https://doi.org/10.1186/s40842–020–00101–8](https://doi.org/10.1186/s40842-020-00101-8).

12. Chiam, P., H.C. Tan, Y.M. Bee, and M. Chandran, Oncogenic osteomalacia –– hypophosphataemic spectrum from "benignancy" to "malignancy"*.* Bone, 2013. 53(1): p. 182–7. <https://doi.org/10.1016/j.bone.2012.11.040>.

13. Cho, S.I., N.Y. Do, S.W. Yu, and J.Y. Choi, Nasal hemangiopericytoma causing oncogenic osteomalacia*.* Clin Exp Otorhinolaryngol, 2012. 5(3): p. 173–6. <https://doi.org/10.3342/ceo.2012.5.3.173>.

14. Clunie, G.P., P.E. Fox, and T.C. Stamp, Four cases of acquired hypophosphataemic ('oncogenic') osteomalacia. Problems of diagnosis, treatment and long–term management*.* Rheumatology (Oxford), 2000. 39(12): p. 1415–21. <https://doi.org/10.1093/rheumatology/39.12.1415>.

15. Ding, J., G. Hu, L. Wang, F. Li, and L. Huo, Increased Activity Due to Fractures Does Not Significantly Affect the Accuracy of 68Ga–DOTATATE PET/CT in the Detection of Culprit Tumor in the Evaluation of Tumor–Induced Osteomalacia*.* Clin Nucl Med, 2018. 43(12): p. 880–886. <https://doi.org/10.1097/RLU.0000000000002290>.

16. Folpe, A.L., J.C. Fanburg–Smith, S.D. Billings, M. Bisceglia, F. Bertoni, J.Y. Cho, M.J. Econs, C.Y. Inwards, S.M. Jan de Beur, T. Mentzel, E. Montgomery, M. Michal, M. Miettinen, S.E. Mills, J.D. Reith, J.X. O'Connell, A.E. Rosenberg, B.P. Rubin, D.E. Sweet, T.N. Vinh, L.E. Wold, B.M. Wehrli, K.E. White, R.J. Zaino, and S.W. Weiss, Most osteomalacia–associated mesenchymal tumors are a single histopathologic entity: an analysis of 32 cases and a comprehensive review of the literature*.* Am J Surg Pathol, 2004. 28(1): p. 1–30. [https://doi.org/10.1097/00000478–200401000–00001](https://doi.org/10.1097/00000478-200401000-00001).

17. Fuentealba, C., D. Pinto, F. Ballesteros, D. Pacheco, O. Boettiger, N. Soto, W. Fernandez, F. Gabler, G. Gonzales, and A.J. Reginato, Oncogenic hypophosphatemic osteomalacia associated with a nasal hemangiopericytoma*.* J Clin Rheumatol, 2003. 9(6): p. 373–9. <https://doi.org/10.1097/01.rhu.0000101906.15276.ed>.

18. Ghosh, S., R. Sinha, R. Bandyopadhyay, and M. Malhotra, Oncogenous osteomalacia*.* J Cancer Res Ther, 2009. 5(3): p. 210–2. [https://doi.org/10.4103/0973–1482.57130](https://doi.org/10.4103/0973-1482.57130).

19. González, G., R. Baudrand, M.F. Sepúlveda, N. Vucetich, F.J. Guarda, P. Villanueva, O. Contreras, A. Villa, F. Salech, L. Toro, L. Michea, and P. Florenzano, Tumor–induced osteomalacia: experience from a South American academic center*.* Osteoporos Int, 2017. 28(7): p. 2187–2193. [https://doi.org/10.1007/s00198–017–4007–2](https://doi.org/10.1007/s00198-017-4007-2).

20. Gonzalez–Compta, X., M. Manos–Pujol, M. Foglia–Fernandez, E. Peral, E. Condom, T. Claveguera, and M. Dicenta–Sousa, Oncogenic osteomalacia: case report and review of head and neck associated tumours*.* J Laryngol Otol, 1998. 112(4): p. 389–92. <https://doi.org/10.1017/s0022215100140551>.

21. Gore, M.O., B.J. Welch, W. Geng, W. Kabbani, N.M. Maalouf, J.E. Zerwekh, O.W. Moe, and K. Sakhaee, Renal phosphate wasting due to tumor–induced osteomalacia: a frequently delayed diagnosis*.* Kidney Int, 2009. 76(3): p. 342–7. <https://doi.org/10.1038/ki.2008.355>.

22. Gresham, M.S., S. Shen, Y.J. Zhang, and K. Gallagher, Anterior Skull Base Glomangioma–Induced Osteomalacia*.* J Neurol Surg Rep, 2017. 78(1): p. e9–e11. [https://doi.org/10.1055/s–0036–1597599](https://doi.org/10.1055/s-0036-1597599).

23. Hana, T., S. Tanaka, H. Nakatomi, M. Shojima, S. Fukumoto, M. Ikemura, and N. Saito, Definitive surgical treatment of osteomalacia induced by skull base tumor and determination of the half–life of serum fibroblast growth factor 23*.* Endocr J, 2017. 64(10): p. 1033–1039. [https://doi.org/10.1507/endocrj.EJ17–0177](https://doi.org/10.1507/endocrj.EJ17-0177).

24. Huang, P.Y., Y.Z. Li, X.M. Yang, B.Q. Yu, L. Guo, Z.T. Guo, and S.S. Li, [Tumor–induced osteomalacia caused by nasal hemangiopericytoma: a case report]*.* Zhonghua Er Bi Yan Hou Tou Jing Wai Ke Za Zhi, 2019. 54(8): p. 615–617. [https://doi.org/10.3760/cma.j.issn.1673–0860.2019.08.012](https://doi.org/10.3760/cma.j.issn.1673-0860.2019.08.012).

25. Inokuchi, G., H. Tanimoto, H. Ishida, T. Sugimoto, M. Yamauchi, A. Miyauchi, and K. Nibu, A paranasal tumor associated with tumor–induced osteomalacia*.* Laryngoscope, 2006. 116(10): p. 1930–3. <https://doi.org/10.1097/01.mlg.0000231295.67060.89>.

26. Jamal, S.A., B.C. Dickson, and I. Radziunas, Tumour induced osteomalacia due to a sinonasal hemangiopericytoma: A case report*.* World J Clin Cases, 2013. 1(1): p. 59–63. <https://doi.org/10.12998/wjcc.v1.i1.59>.

27. Jefferis, A.F., P.C. Taylor, and G.P. Walsh–Waring, Tumour–associated hypophosphataemic osteomalacia occurring in a patient with an odontogenic tumour of the maxilla*.* J Laryngol Otol, 1985. 99(10): p. 1011–7. <https://doi.org/10.1017/s0022215100098091>.

28. John, M.R., H. Wickert, K. Zaar, K.B. Jonsson, A. Grauer, P. Ruppersberger, H. Schmidt–Gayk, H. Murer, R. Ziegler, and E. Blind, A case of neuroendocrine oncogenic osteomalacia associated with a PHEX and fibroblast growth factor–23 expressing sinusidal malignant schwannoma*.* Bone, 2001. 29(4): p. 393–402. [https://doi.org/10.1016/s8756–3282(01)00586–5](https://doi.org/10.1016/s8756-3282(01)00586-5).

29. John, J.R., J. Hephzibah, R. Oommen, N. Shanthly, and D. Mathew, Ga–68 DOTATATE Positron Emission Tomography–Computed Tomography Imaging in Oncogenic Osteomalacia: Experience from a Tertiary Level Hospital in South India*.* Indian J Nucl Med, 2019. 34(3): p. 188–193. [https://doi.org/10.4103/ijnm.IJNM–14–19](https://doi.org/10.4103/ijnm.IJNM_14_19).

30. Kabtouri, H., R. Karmali, L. Spinato, M. Khamaktchian, and P. Bisschop, Oncogenic osteomalacia induced by a sinonasal tumour: case report and review*.* B–ENT, 2014. 10(2): p. 149–55

31. Kane, S.V., A. Kakkar, N. Oza, E. Sridhar, and P.S. Pai, Phosphaturic mesenchymal tumor of the nasal cavity and paranasal sinuses: A clinical curiosity presenting a diagnostic challenge*.* Auris Nasus Larynx, 2018. 45(2): p. 377–383. <https://doi.org/10.1016/j.anl.2017.05.006>.

32. Kawai, Y., S. Morimoto, K. Sakaguchi, H. Yoshino, T. Yotsui, S. Hirota, H. Inohara, T. Nakagawa, K. Hattori, T. Kubo, J. Yang, N. Fujiwara, and T. Ogihara, Oncogenic osteomalacia secondary to nasal tumor with decreased urinary excretion of cAMP*.* J Bone Miner Metab, 2001. 19(1): p. 61–4. <https://doi.org/10.1007/s007740170062>.

33. Kenealy, H., I. Holdaway, and A. Grey, Occult nasal sinus tumours causing oncogenic osteomalacia*.* Eur J Intern Med, 2008. 19(7): p. 516–9. <https://doi.org/10.1016/j.ejim.2008.01.011>.

34. Kominek, P., I. Starek, M. Geierova, P. Matousek, and K. Zelenik, Phosphaturic mesenchymal tumour of the sinonasal area: case report and review of the literature*.* Head Neck Oncol, 2011. 3: p. 16. [https://doi.org/10.1186/1758–3284–3–16](https://doi.org/10.1186/1758-3284-3-16).

35. Koriyama, N., K. Nishimoto, T. Kodama, M. Nakazaki, Y. Kurono, H. Yoshida, and C. Tei, Oncogenic osteomalacia in a case with a maxillary sinus mesenchymal tumor*.* Am J Med Sci, 2006. 332(3): p. 142–7. [https://doi.org/10.1097/00000441–200609000–00010](https://doi.org/10.1097/00000441-200609000-00010).

36. Kurien, R., V. Rupa, and M. Thomas, Varied presentation of sinonasal phosphaturic mesenchymal tumour: report of a case series with follow–up*.* Eur Arch Otorhinolaryngol, 2019. 276(6): p. 1677–1684. [https://doi.org/10.1007/s00405–019–05341–8](https://doi.org/10.1007/s00405-019-05341-8).

37. Lee, H.K., W.W. Sung, P. Solodnik, and M. Shimshi, Bone scan in tumor–induced osteomalacia*.* J Nucl Med, 1995. 36(2): p. 247–9

38. Lee, G.G., H.J. Dhong, Y.S. Park, and Y.H. Ko, Sinonasal glomangiopericytoma causing oncogenic osteomalacia*.* Clin Exp Otorhinolaryngol, 2014. 7(2): p. 145–8. <https://doi.org/10.3342/ceo.2014.7.2.145>.

39. Lee, J.Y., H.S. Park, S. Han, J.K. Lim, N. Hong, S.I. Park, and Y. Rhee, Localization of Oncogenic Osteomalacia by Systemic Venous Sampling of Fibroblast Growth Factor 23*.* Yonsei Med J, 2017. 58(5): p. 981–987. <https://doi.org/10.3349/ymj.2017.58.5.981>.

40. Li, J., Y. Huang, F. Yang, Q. Zhang, D. Chen, and Q. Wang, Sinonasal hemangiopericytoma caused hypophosphatemic osteomalacia: A case report*.* Medicine (Baltimore), 2018. 97(52): p. e13849. <https://doi.org/10.1097/MD.0000000000013849>.

41. Massaccesi, M., F. Micciche, M. Rigante, G. Petrone, E. Lepre, M.A. Gambacorta, and V. Valentini, Successful Treatment of Tumor–Induced Osteomalacia by Multidisciplinary Therapy with Radiation to Intracranial Fibromyxoid Tumor*.* Case Rep Endocrinol, 2021. 2021: p. 8841259. <https://doi.org/10.1155/2021/8841259>.

42. Mathis, D.A., E.J. Stehel, Jr., J.E. Beshay, B.E. Mickey, A.L. Folpe, and J. Raisanen, Intracranial phosphaturic mesenchymal tumors: report of 2 cases*.* J Neurosurg, 2013. 118(4): p. 903–7. <https://doi.org/10.3171/2012.12.JNS12598>.

43. Mok, Y., J.C. Lee, J.H. Lum, and F. Petersson, From epistaxis to bone pain–report of two cases illustrating the clinicopathological spectrum of phosphaturic mesenchymal tumour with fibroblast growth factor receptor 1 immunohistochemical and cytogenetic analyses*.* Histopathology, 2016. 68(6): p. 925–30. <https://doi.org/10.1111/his.12872>.

44. Muniz, C.R., G.A.M. Bezerra, V.C. da Silva, P.M.F. Aguiar, G. Gerson, C.B. D'Alva, and A.A.A. Nunes, Ethmoid glomangioma and oncogenic osteomalacia: a case report*.* J Med Case Rep, 2021. 15(1): p. 348. [https://doi.org/10.1186/s13256–021–02916–0](https://doi.org/10.1186/s13256-021-02916-0).

45. Ohashi, K., T. Ohnishi, T. Ishikawa, H. Tani, K. Uesugi, and M. Takagi, Oncogenic osteomalacia presenting as bilateral stress fractures of the tibia*.* Skeletal Radiol, 1999. 28(1): p. 46–8. <https://doi.org/10.1007/s002560050471>.

46. Okamiya, T., K. Takahashi, H. Kamada, J. Hirato, T. Motoi, S. Fukumoto, and K. Chikamatsu, Oncogenic osteomalacia caused by an occult paranasal sinus tumor*.* Auris Nasus Larynx, 2015. 42(2): p. 167–9. <https://doi.org/10.1016/j.anl.2014.10.001>.

47. Pal, R., S.K. Bhadada, A. Singhare, A. Bhansali, S. Kamalanathan, M. Chadha, P. Chauhan, A. Sood, V. Dhiman, D.C. Sharma, U.N. Saikia, D. Chatterjee, and V. Agashe, Tumor–induced osteomalacia: experience from three tertiary care centers in India*.* Endocr Connect, 2019. 8(3): p. 266–276. [https://doi.org/10.1530/EC–18–0552](https://doi.org/10.1530/EC-18-0552).

48. Papierska, L., J.B. Cwikla, W. Misiorowski, M. Rabijewski, K. Sikora, and H. Wanyura, Unusual case of phosphaturic mesenchymal tumor*.* Pol Arch Med Wewn, 2013. 123(5): p. 255–6

49. Papotti, M., M.P. Foschini, G. Isaia, G. Rizzi, C.M. Betts, and V. Eusebi, Hypophosphatemic oncogenic osteomalacia: report of three new cases*.* Tumori, 1988. 74(5): p. 599–607. <https://doi.org/10.1177/030089168807400519>.

50. Parshwanath, H.A., P.R. Kulkarni, R. Rao, S.K. Joshi, and P. Patil, Phosphaturic mesenchymal tumor of ethmoid sinus*.* Indian J Pathol Microbiol, 2010. 53(2): p. 384–5. [https://doi.org/10.4103/0377–4929.64317](https://doi.org/10.4103/0377-4929.64317).

51. Pedrazzoli, M., G. Colletti, M. Ferrari, G. Rossetti, L. Moneghini, and L. Autelitano, Mesenchymal phosphaturic neoplasm in the maxillary sinus: a case report*.* Int J Oral Maxillofac Surg, 2010. 39(10): p. 1027–32. <https://doi.org/10.1016/j.ijom.2010.04.039>.

52. Peterson, N.R., D.J. Summerlin, and S.R. Cordes, Multiple phosphaturic mesenchymal tumors associated with oncogenic osteomalacia: case report and review of the literature*.* Ear Nose Throat J, 2010. 89(6): p. E11–5

53. Ray, S., P.P. Chakraborty, K. Biswas, S. Ghosh, S. Mukhopadhyay, and S. Chowdhury, A case of oncogenic osteomalacia due to occult nasal sinus tumor*.* Clin Cases Miner Bone Metab, 2015. 12(1): p. 65–8. <https://doi.org/10.11138/ccmbm/2015.12.1.065>.

54. Renton, P. and D.J.S.R. Shaw, Hypophosphatemic osteomalacia secondary to vascular tumors of bone and soft tissue*.* 1976. 1(1): p. 21–24

55. Rigante, M., A. Loperfido, and G. Paludetti, Oncogenic Osteomalacia with Elevated Fibroblast Growth Factor 23: A Rare Case of Paranasal Sinus Tumor Onset*.* Cureus, 2019. 11(6): p. e4919. <https://doi.org/10.7759/cureus.4919>.

56. Sandhu, F.A. and R.L. Martuza, Craniofacial hemangiopericytoma associated with oncogenic osteomalacia: case report*.* J Neurooncol, 2000. 46(3): p. 241–7. <https://doi.org/10.1023/a:1006352106762>.

57. Seshadri, M.S., C.J. Cornish, R.S. Mason, and S. Posen, Parathyroid hormone–like bioactivity in tumours from patients with oncogenic osteomalacia*.* Clin Endocrinol (Oxf), 1985. 23(6): p. 689–97. [https://doi.org/10.1111/j.1365–2265.1985.tb01130.x](https://doi.org/10.1111/j.1365-2265.1985.tb01130.x).

58. Shah, R., A.R. Lila, R.S. Jadhav, V. Patil, A. Mahajan, S. Sonawane, P. Thadani, A. Dcruz, P. Pai, M. Bal, S. Kane, N. Shah, and T. Bandgar, Tumor induced osteomalacia in head and neck region: single center experience and systematic review*.* Endocr Connect, 2019. 8(10): p. 1330–1353. [https://doi.org/10.1530/EC–19–0341](https://doi.org/10.1530/EC-19-0341).

59. Shelekhova, K.V., D.V. Kazakov, and M. Michal, Sinonasal phosphaturic mesenchymal tumor (mixed connective tissue variant): report of 2 cases*.* Am J Surg Pathol, 2010. 34(4): p. 596–7. <https://doi.org/10.1097/PAS.0b013e3181d594fa>.

60. Sweet, R.A., J.L. Males, A.J. Hamstra, and H.F. DeLuca, Vitamin D metabolite levels in oncogenic osteomalacia*.* Ann Intern Med, 1980. 93(2): p. 279–80. [https://doi.org/10.7326/0003–4819–93–2–279](https://doi.org/10.7326/0003-4819-93-2-279).

61. Tang, R., S. Mao, H. Lin, H.B. Ye, D.W. Li, Z.N. Chen, K.M. Su, and W.T. Zhang, Surgical Treatment and Outcomes for Sinonasal and Skull Base Phosphaturic Mesenchymal Tumors*.* Otolaryngol Head Neck Surg, 2020. 162(5): p. 674–682. <https://doi.org/10.1177/0194599820904055>.

62. Then, C., E. Asbach, H. Bartsch, N. Thon, C. Betz, M. Reincke, and R. Schmidmaier, Fibroblast Growth Factor 23–Producing Phosphaturic Mesenchymal Tumor with Extraordinary Morphology Causing Oncogenic Osteomalacia*.* Medicina (Kaunas), 2020. 56(1). <https://doi.org/10.3390/medicina56010034>.

63. Villepelet, A., O. Casiraghi, S. Temam, and A. Moya–Plana, Ethmoid tumor and oncogenic osteomalacia: Case report and review of the literature*.* Eur Ann Otorhinolaryngol Head Neck Dis, 2018. 135(5): p. 365–369. <https://doi.org/10.1016/j.anorl.2018.07.001>.

64. Xian–Ling, W., B. Jian–Ming, Z. Wen–Wen, L. Zhao–Hui, D. Jing–Tao, L. Ju–Ming, and M. Yi–Ming, Osteomalacia caused by tumors in facies cranii mimicking rheumatoid arthritis*.* Rheumatol Int, 2012. 32(8): p. 2573–6. [https://doi.org/10.1007/s00296–011–2018–4](https://doi.org/10.1007/s00296-011-2018-4).

65. Wang, T., S. Qu, and L. Ye, [Sinonasal glomus tumour–induced osteomalacia: a case report]*.* Zhonghua Er Bi Yan Hou Tou Jing Wai Ke Za Zhi, 2014. 49(8): p. 691–2

66. Weidner, N., R.S. Bar, D. Weiss, and M.P. Strottmann, Neoplastic pathology of oncogenic osteomalacia/rickets*.* Cancer, 1985. 55(8): p. 1691–705. [https://doi.org/10.1002/1097–0142(19850415)55:8](https://doi.org/10.1002/1097-0142(19850415)55:8)<1691::aid–cncr2820550814>3.0.co;2–s.

67. Yu, W.J., J.W. He, W.Z. Fu, C. Wang, and Z.L. Zhang, Reports of 17 Chinese patients with tumor–induced osteomalacia*.* J Bone Miner Metab, 2017. 35(3): p. 298–307. [https://doi.org/10.1007/s00774–016–0756–9](https://doi.org/10.1007/s00774-016-0756-9).

68. Zhu, Z., W. Xia, F. Qi, W. Wang, X. Wang, Y. Zha, F. Li, H. Jing, P. Wang, and W. Lv, Clinical Characteristics and Surgical Outcomes of Sinonasal Lesions Associated With Tumor–Induced Osteomalacia*.* Otolaryngol Head Neck Surg, 2021. 165(1): p. 223–231. <https://doi.org/10.1177/0194599820975432>.

69. Zuo, Q.Y., H. Wang, W. Li, X.H. Niu, Y.H. Huang, J. Chen, Y.H. You, B.Y. Liu, A.M. Cui, and W. Deng, Treatment and outcomes of tumor–induced osteomalacia associated with phosphaturic mesenchymal tumors: retrospective review of 12 patients*.* BMC Musculoskelet Disord, 2017. 18(1): p. 403. [https://doi.org/10.1186/s12891–017–1756–1](https://doi.org/10.1186/s12891-017-1756-1).

70. Jiang, Y., W.B. Xia, X.P. Xing, B.C. Silva, M. Li, O. Wang, H.B. Zhang, F. Li, H.L. Jing, D.R. Zhong, J. Jin, P. Gao, L. Zhou, F. Qi, W. Yu, J.P. Bilezikian, and X.W. Meng, Tumor-induced osteomalacia: an important cause of adult-onset hypophosphatemic osteomalacia in China: Report of 39 cases and review of the literature*.* J Bone Miner Res, 2012. 27(9): p. 1967-75. <https://doi.org/10.1002/jbmr.1642>.
